# Biodegradable Blends of Grafted Dextrin with PLGA-*block*-PEG Copolymer as a Carrier for Controlled Release of Herbicides into Soil

**DOI:** 10.3390/ma13040832

**Published:** 2020-02-12

**Authors:** Kamila Lewicka, Piotr Rychter, Małgorzata Pastusiak, Henryk Janeczek, Piotr Dobrzynski

**Affiliations:** 1 Faculty of Science and Technology, Jan Dlugosz University in Czestochowa, 13/15 Armii Krajowej Av., 42-200 Czestochowa, Poland; kamila.lewicka@ujd.edu.pl (K.L.); p.rychter@ujd.edu.pl (P.R.); 2 Centre of Polymer and Carbon Materials, Polish Academy of Sciences, 34 Curie-Sklodowskiej Str., 41-819 Zabrze, Poland; mpastusiak@cmpw-pan.edu.pl (M.P.); hjaneczek@cmpw-pan.edu.pl (H.J.)

**Keywords:** grafted dextrin, grafted maltodextrin, PLGA, PEG, controlled release, metazachlor, pendimethain, biodegradable polymers

## Abstract

The presented work aimed to test influence of poly(L-lactide-co-glycolide)-*block*-poly (ethylene oxide) copolymer modification by blending with grafted dextrin or maltodextrin on the course of degradation in soil and the usefulness of such material as a matrix in the controlled release of herbicides. The modification should be to obtain homogenous blends with better susceptibility to enzymatic degradation. Among all tested blends, which were proposed as a carrier for potential use in the controlled release of plant protection agents, PLGA-*block*-PEG copolymer blended with grafted dextrin yielded very promising results for their future applications, and what is very importantly proposed formulations provide herbicides in unchanged form into soil within few months of release. The modification PLAGA/PEG copolymer by blending with modificated dextrins affects the improvement of the release profile. The weekly release rates for both selected herbicides (metazachlor and pendimethalin) were constant for a period of 12 weeks. Enzymatic degradation of modified dextrin combined with leaching of the degradation products into medium caused significant erosion of the polymer matrix, thereby leading to acceleration of water diffusion into the polymer matrix and allowing for easier leaching of herbicides outside the matrix.

## 1. Introduction

Conventional formulations of agrochemicals can contaminate the environment, in particular for intensive cropping. Hence, there is a need for controlled release formulations of agrochemicals such as polysaccharides to reduce pollution and health hazards. Natural polymers are gaining considerable acceptance over synthetic polymers as controlled-release devices because of their eco-friendly nature, cost-effectiveness, easy availability, and biodegradability [1,2]. Starch is abundant in nature and is one of the most important carbohydrates. Biodegradability of starch has attracted attention in scientific and industrial fields, and it has many uses. However, raw starch has some defects that limit its industrial applications; therefore, modifications are needed. Grafting is an important technique for modifying the properties of polymers. Chemical modification of starch by graft copolymerization makes starch and synthetic polymer bind together rather than exist merely as a physical mixture. Hence, synthetic monomers and polymers were used to modify the properties of raw starch, and semi-synthetic polymers were obtained, thereby increasing application areas and attracting the attention of researchers [3]. Graft copolymerization provides a tool for researchers to incorporate targeted properties in the backbone of polymers for specialized applications without affecting their biodegradability. It is a technique to hybridize synthetic and natural polymers and enable fundamental investigation of their structure–property relationships. Properties such as melting point, glass transition temperature, solubility, permeability, chemical reactivity, and elasticity can be modified through graft copolymerization according to the specific requirements [4,5]. In recent years, the modification of chemical and physical properties of natural polymers and their derivatives through graft copolymerization has attracted much attention [6,7,8,9,10,11,12]. Graft copolymerization of various vinyl monomers onto different polysaccharides has been reported by various researchers. Because polysaccharides occur abundantly in nature, this class of polymers holds special significance for scientists.

The starch graft-copolymer is a representative of the modification of starch molecules by the chemical method. Starch graft-copolymers such as starch-g-polystyrene (St-g-PS) [13], starch-g-methacrylonitrile [14], starch-g-polyvinyl alcohol [15], and starch-g-acrylonitrile [16], have been synthesized by generating free radicals on the surface of starch granules and the copolymerization of these free radicals with the respective vinyl monomers. However, these copolymers with vinyl polymer branches also have limited biodegradability because of the presence of the nondegradable part of the polymer, although their properties are acceptable for applications. Another effort in chemical modification of starch is grafting starch with biodegradable polymers [17], such as poly (e-caprolactone (PCL) and PLA.

Graft-copolymers such as starch-g-PCL and starch-g-PLA can be completely biodegraded by bacteria or under natural conditions and have improved mechanical performance. Therefore, graft-copolymers can be used directly as thermoplastics or as a compatibilizer in starch-PCL or starch-PLA blend composites [18]. In the present study, by using the ‘graft-from’ strategy, we grafted PCL onto the surface of dextrin and maltodextrin by ring-opening polymerization. Thus far, dextrins have been grafted with poly (N-vinyl caprolactam) [19], poly (butyl acrylate-co-styrene) [20], and polylactide [21]. Similar polysaccharide-dextran was grafted with polycaprolactone [22]. We previously investigated a blocked terpolymer with a chain composed of two blocks, namely L-lactide/glycolide copolymer block and polyethylene glycol (PEG) block [23], where we demonstrated the effect of comparatively small content of PEG blocks on strong acceleration of degradation in activated sludge or in soil. Moreover, we demonstrated the usefulness of these terpolymers as a biodegradable carrier for the controlled release of selected pesticides. However, in order to achieve an adequate release rate that ensures that the entire amount of herbicides tested is delivered to the soil within about 4 months (average vegetation period of the plants to be harvested), it is necessary to develop a carrier that will allow faster degradation by enzymes. We wanted to achieve this effect by modifying the previously used polymeric material by grafting with maltodextrin and dextrin. This modification can obtain miscible blends with better susceptibility to uniform enzymatic degradation. Efforts were made to avoid the use of volatile solvents in the work, which is why both PLAGA-*block*-PEG terpolymer synthesis and dextrins modification were carried out in bulk. The obtained results are presented in this paper.

## 2. Materials and Methods

### 2.1. Materials

#### 2.1.1. Monomers, Initiators, Catalyst, Herbicides

The reagent used to grafting were ε-caprolactone (Acros Organics, Geel, Belgium) purified using reduced pressure vacuum distillation, dextrin from corn (dextrose equivalent DE; 9-12) and maltodextrin (DE; 12-14) which were purchased from Sigma-Aldrich and used as received. Zinc acetylacetonate Zn(acac)2—catalyst (Sigma-Aldrich, Steinheim, Germany) were used as received.

L-lactide and glycolide (Glaco Ltd., Beijing, China) were purified by recrystallization from ethyl acetate and was dried in a vacuum oven at room temperature. Polyethylene glycol—PEG 4600 (Aldrich Corp., Steinheim, Germany) was dried in a vacuum oven at room temperature. Zirconium (IV) acetylacetonate Zr(Acac)4 (Aldrich Corp., Steinheim, Germany) was catalyst of the copolymerization were used as received. Metazachlor and pendimethalin (Sigma-Aldrich) were used as received. Methanol, chloroform, and other organic solvents (Chempur, Piekary Slaskie, Poland) were used as received.

#### 2.1.2. Soil and Activated Sludge

Soil basic properties: granulometric composition of soil equaled to 77% sand; 19% dust; and loam, the organic carbon content, of approx. 1.8%, with the active acidity value 5.9 (concentration of H^+^ in soil solution). Activated sludge was derived from sewage treatment plant “WARTA” in Czestochowa. The most important properties are: total dry solids 6–12%; N 1.6–6.0% d.m.; P2O5 1.5–4.0% d.m.; K_2_O 1–3% d.m. and pH 7.2–7.5).

### 2.2. Methods of Synthesis

#### 2.2.1. Synthesis of L-Lactide/Glycolide/PEG Terpolymers (PLAGA-PEG)

The terpolymers PLAGA-PEG were synthesized by ROP mechanism. The copolymerization of L-lactide with glycolide was conducted in bulk with an Zr(Acac)4 as catalyst with a molar ratio of 1/800 and with use of polyethylene glycols—PEG 4600 as macroinitiator. The molar ratio of L-lactide to glycolide in the output reaction mixture was 85:15 and was practically this same in the final terpolymer. The final contain of PEG in terpolymer was 10 wt %, glycolidyl—12 wt % and L-lactidyl about 78 wt %. The average molecular weight of the obtained terpolymer was about 60,000 g/mol with molecular dispersion Mw/Mn = 2.8 The detailed course of the synthesis and its conditions are described in our earlier publication [23], where also shown the usefulness of the terpolymer as a matrix used in long-term controlled release of herbicides into soil.

#### 2.2.2. Synthesis of Dextrin-g-PCL and Maltodextrin-g-PCL

**Scheme 1:**
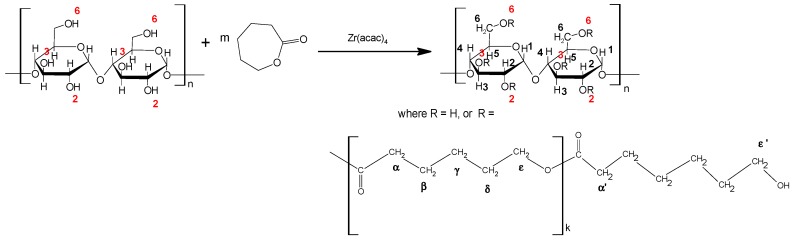
Course of the conducted grafting reaction.

Copolymerization of dextrin or maltodextrin with ε-caprolactone (CL) (Scheme 1) was carried out in bulk. The weighed amount of ε-caprolactone, dextrin or maltodextrin and zirconium (IV) acetylacetonate as the catalyst (molar ratio catalyst/ε-caprolatone as 1:600) were charged into dried glass reactor and heated in an oil bath at 130 °C for 48 h or 72 h in an argon atmosphere with constant stirring (Scheme 1, Table 1). After the selected reaction time we removed the unreacted CL, free polycaprolactone (PCL) and the most of used catalyst by extraction with chloroform. The remaining insoluble gel fraction was then dried in vacuum, at 40 °C. The dry product after grinding was washed with water to remove eventual residues of ungrafted polysaccharides. Obtained dextrin-g-PCL or maltodextrin-g-PCL was left to dry in vacuum drier for constant mass.

#### 2.2.3. Preparation of the PLAGA-Block-PEG/Dextrin-g-PCL or PLAGA-Block-PEG/Maltodextrin-g-PCL Blends’ Films

In brief, different aliquots of dextrin-g-PCL and maltodextrin-g-PCL solutions (10 wt % in dry DMSO) were slowly added to 20 mL of PLGA-block-PEG terpolymer solutions (15 wt % in chloroform) to obtain 30, and 50 wt % in the grafted polysacharide/PLAGA-b-PEG blend. In case of preparation of blends with unmodified polysaccharides, the terpolymer was dissolved in DMSO. The final blends’ films were prepared by pouring these mixtures into Teflon dishes, drying initially for 48 h in air and then under vacuum at 40 °C to a constant mass, so that the remaining solvent evaporates. Then the film samples were cut into small disks: diameter 18 mm; thickness about 140–160 μm.

### 2.3. Measurements

#### 2.3.1. Nuclear Magnetic Resonance (NMR) Spectroscopy

Nuclear magnetic resonance analysis was carried out at 600 MHz with the Advance II Bruker Ultrashield Plus Spectrometer (Bruker Corporation, Anaheim, CA, USA). NMR spectra were recorded with 32 scans, a 2.65 s acquisition time, and an 11 ms pulse at 26 °C, using DMSO-d6 as solvent. The chemical shifts were measured in ppm relative to the tetramethylsilane signal.

#### 2.3.2. Thermal Properties

Thermal properties, such as glass transition temperatures and heats of melting and crystallization of obtained copolymers and blends were investigated by differential scanning calorimetry (DuPont, Newark, DE, USA) calibrated with gallium and indium. The glass transition temperature was determined with heating and cooling rate of 20 °C/min in the range between −100 °C and 220 °C, according to the ASTM E 1356-08 standard. The measurements were conducted after prior amorphization of the samples by melting and then very fast cooling in liquid nitrogen. Blend samples, for deeper analysis, were also tested using different heating rates.

#### 2.3.3. Fourier Transformation Infrared Spectra (FTIR)

FTIR spectra were determined using Nexus Nicolete spectrophotometer (Madison, WIS., USA) in KBr discs in the range of 4000–400 cm^−1^ at 32 scans of samples.

#### 2.3.4. UV–Vis Spectroscopy

A Thermo Scientific™ Helios Gamma Spectrophotometer (Thermo Fisher, Karlsruhe, Germany) was used for analysis based on the method UV–Vis spectroscopy for release kinetics tests in various media, such as water and soil. Measurements of concentrations of herbicide solutions were carried out on the basis of calibration curves (Supported Information, Figure S1).

#### 2.3.5. Scanning Electron Microscopy (SEM) Observation

A Tescan model VEGA 3SBU scanning electron microscope (SEM) (Tescan Orsay Holding, Brno, Czech Republic) was used for evaluation of miscibility of produced blends. Both the surface appearance of the samples and their cross-section were tested. In addition, the surface deterioration of samples after period of exposure to degradation medium were examined. The applied accelerating voltage for measurement was 1–3 kV, under low vacuum by using the secondary electron mode and the samples were not coated with a conductive layer.

### 2.4. Studies of the Blends’ Degradation and Herbicides’ Release

#### 2.4.1. Degradation in Water

Degradation of blends was carried out in distilled water. Each film sample, in triplicate, was immersed in a vial filled with 10 mL of distilled water and placed at room temperature. After 0.5, 1, 1.5, 2, 2.5, and 3 month of experiment time. The film samples were retrieved for the monitor of the degradation process. The disks of each blend after each degradation time were vacuum-dried at room temperature and then subjected to detailed testing.

#### 2.4.2. Degradation in Soil

For degradation studies conducted in soil, the previously prepared disks formed with the selected blends were packed in pockets of polyethylene mesh and placed in flower pots with soil. The flower pots were kept under controlled conditions with a constant humidity content at the level required for the plants (70% field water capacity), temperature (22 ± 2 °C) and light intensity (7000 lux), which were maintained in the condition imitating cycles of day/night—16/8 h. After 0.5, 1, 1.5, 2, 2.5, and 3 month of experiment time, the films samples were retrieved for the monitor of the degradation process. The disks of each blend after each degradation time were washed with distilled water, vacuum-dried at room temperature and then subjected to detailed testing.

#### 2.4.3. Degradation in Activated Sludge

The disks were placed in a vial filled with 10 mL of activated sludge with properties were typical for urban waste water treatment plant. Activated sludge was exchanged for fresh every 14 days. Degradations were carried out at room temperature by monitoring the microbial viability during the process, dry matter, sedimentation, sludge index, visual assessment using an electron microscope, and pH analysis. Similarly, after 0.5, 1, 1.5, 2, 2.5, and 3 month of experiment time, the disks of each blend after each degradation time were washed with distilled water, vacuum-dried at room temperature and then subjected to detailed testing.

#### 2.4.4. Water Uptake and Weight Loss Measurements

Each of sample was weighed before and after degradation using an electronic balance (0.1 mg precision) (Radwag, Radom, Poland). Water uptake and weight loss was calculated using the relationship

(1) $$Water\;uptake\;\left(\%\right)=\frac{\left(W_{wet}-W_{dry}\right)}{\left(W_{dry}\right)}\;\times 100\%$$

(2) $$Weight\;loss\;\left(\%\right)=\frac{\left(W_{0}-W_{dry}\right)}{\left(W_{0}\right)}\;\times 100\%$$

where *W_wet_* means the wet weight after wiping, *W_dry_* the dry weight after vacuum drying, and *W*_0_ the initial weight.

#### 2.4.5. Release of Herbicides into Water and Soil

Each types of samples with metazachlor or pendimethalin (10 and 20 wt %) were placed in closed bottles with 10 mL of distilled water. The medium was exchanged at each time of measurement. For tests on the release of herbicides into the soil, similar samples were placed in a flowerpot with soil (eight disks at equal intervals in one flowerpot). The samples were incubated at room temperature for 3 months. At regular time periods, the pesticide concentration was measured. The absorbance level of released herbicides was analysed by UV–Vis spectroscopy for metazachlor at λ = 228 nm and for pendimethalin at 238 nm.

The kinetics of herbicide release into the soil was analyzed by placing in the middle of a pot (250 mL volume with standardized soil) a previously carefully weighed polymeric film. During the test, constant humidity was kept (regular watering of pots) and constant temperature (22 °C). After the incubation time, the polymeric films were extracted, washed with distilled water, dissolved, and analyzed according to the methods described above. The herbicides content was determined on the basis of the analysis of the difference between the initial amount in the carrier and the remaining amount.

## 3. Results and Discussion

### 3.1. Synthesis and Characterization of Obtained Grafted Dextrin and Grafted Maltodextrin

During the grafting reactions of dextrin or maltodextrin, we used various amounts of ε-caprolactone excess, as well as differentiation of the reaction time (Scheme 1, Table 1). On the basis of preliminary tests, zirconium (IV) acetylacetonate was selected as the most effective and nontoxic catalyst of the conducted reaction. In addition, it is known that this complex is not sensitive to any contaminants or traces of water and tends to be inert toward various chemicals at low temperatures, which allows polymerization of impure monomers, even in presence of organic acids [24,25].

**Figure 1:**
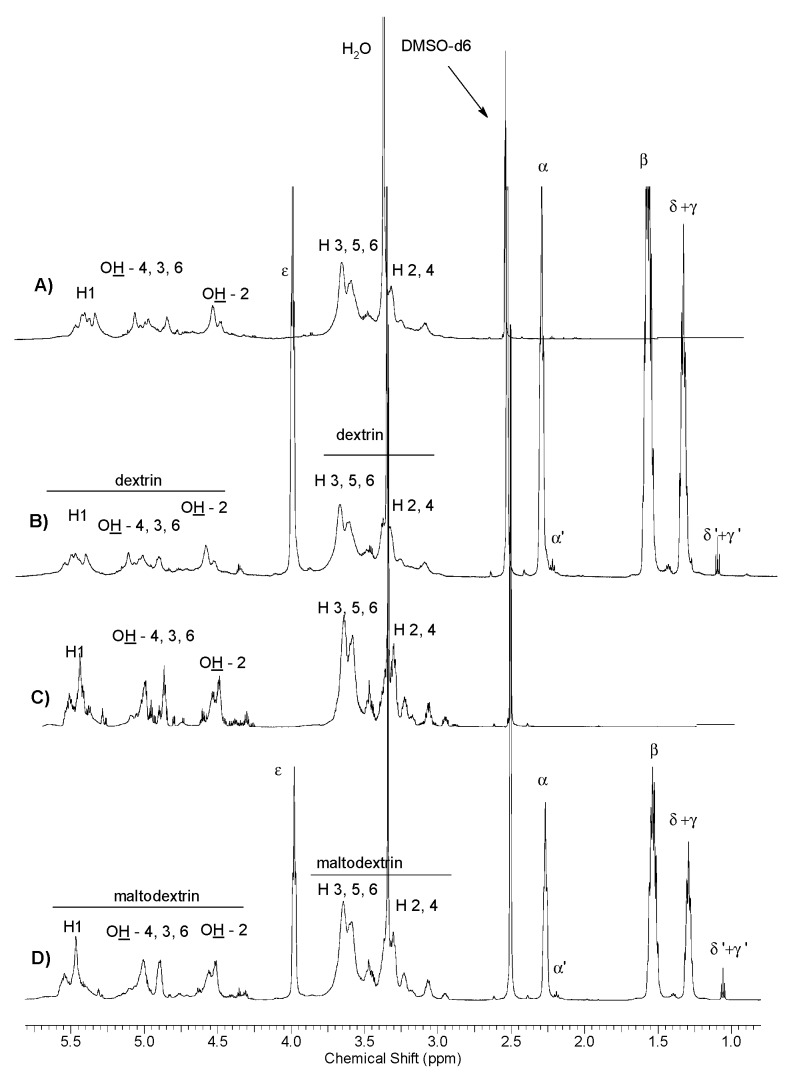
1H NMR spectra of: (**A**) dextrin, (**B**) dextrin-g-PCL with weight fraction of dextrin—31 wt %, (**C**) maltodextrin, and (**D**) maltodextrin-g-PCL with weight fraction of maltodextrin—38 wt %.

This condition, despite the simple way of the conducting reaction, could obtain products with different degrees of substitution of the hydroxyl groups of the polysaccharide chain as well as different contents (lengths) of the grafted caproyl chains (Table 1). In all the conducted reactions of grafting of dextrin and maltodextrin, a high conversion of ε-caprolactone—above 90%—was obtained. The progress of the reaction, the composition of the copolymer, and the degree of substitution were measured based on the proton NMR spectra (Figure 1). The obtained spectra of PCL grafts of dextrin and maltodextrin were similar to those of the analogous previously presented spectra of starch grafted with PCL [17,26], and in signal regions assigned to dextrin grafted with L-lactide [21]. However, during literature review, we noted that there are fundamental discrepancies in the assignment of signals to dextrin or starch protons. After analyzing the literature describing the 1H NMR studies of oligosaccharides, grafted dextrins, and starch, as well as our research, we assigned the signals essentially according to the proposed method in most published studies in this context [19,20,27,28]. The obtained proton NMR spectra of the dextrin-g-PCL and maltodextrin-g-PCL with proper assignment are presented in Figure 1. The peaks of the PCL CH_2_ units are present in the range of δ 1.2–4 ppm and imply that the ring-opening polymerization reaction of CL occurred. Resonances from the dextrin and maltodextrin peaks are observed as relatively small, broad peaks in the region δ 2.9–3.8 and 4.4–5.6 ppm. Numbers H1–H6 denote the protons of CH and CH_2_ groups of dextrin and the Greek symbols indicate the protons in the CL unit. It is apparent that H2, H4 peaks of dextrin were observed at δ = 3.0–3.2 ppm, while H3, H5, and H6 peaks of dextrin were found at δ = 3.4–3.7 ppm. The hydroxyl protons of dextrin (OH2, OH3, and OH6) were observed at δ = 4.3–5.2 ppm. The anomeric hydrogen H1 was obtained at δ = 5.3–5.6 ppm.

Analogous to previously described grafting of dextran with ε-caprolactone [22], to determine relative weight fraction of the glucopyranosyl ring to CL units in the obtained graft copolymer (dextrin/CL), we used equation.

(3) $$R_{dex}=\frac{162\;I\left(H1\right)}{162\;I\left(H1\right)+\;\frac{114}{2}\;I(\varepsilon )}\;\times \;100\;\mathrm{wt}\;\%\;$$

where *R_dex_*—percentage weight ratio of dextrin /CL; *I*(*H*1)—the values of the integrals of the dextrin’s anomeric hydrogen, well separated from the other proton signals (3.2–3.7 ppm) in spectrum obtained from 1H NMR; and *I*(*ε*)—the values of the integrals of polycaprolactone ε CH_2_ groups protons.

The relative weight fraction for grafted dextrin between 75% and 32% was designated, which was dependent on the starting composition of the reaction mixture. The actual content of the grafted dextrin in the copolymer was always greater by at least 20% than the dextrin content in started reaction mixture; this indicates that a significant part of monomeric caprolactone was polymerized to homo-polycaprolactone. This homopolymer was removed by extraction. The observed NMR signals of the end caproyl chain CH_2_ groups allow to estimate their average length. It was quite surprising that the average length of caproyl block was slightly smaller for copolymers with maltodextrin (Table 1, samples 1–3) than for the dextrin copolymers, although the caproyl content was larger (Table 1, samples 6–9). The degree of attachment of the caproyl chains to saccharide units is substantially greater, which indicates that the reaction of the maltodextrin hydroxyl groups was more efficient than reaction with dextrin. This observed effect proves that the polymerization of caprolactone with dextrin occurs at the interface phase and the growth of caproyl chains occurred mainly at the primary group OH of saccharide units. Thus, relatively long grafting chains were obtained. With the increase in the amount of ε-caprolactone in the initial reaction mixture, the number of grafted caproyl units only slightly increased, but the average length of the caproyl block decreased considerably. This indicates that, under these conditions, the number of substituted hydroxyl groups of the glucopiranosyl rings increases and thus the amount of grafted PCL chains. The FTIR spectra of the dextrin-g-PCL copolymer, dextrin, and polycaprolactone PCL are shown in Figure S2 (Supporting information). The IR spectrum of dextrin has been reported in detail earlier [19,29]. The main difference between the spectrum of the copolymer dextrin-g-PCL with a small ratio of dextrin (Figure S2A) and the spectrum of nongrafted dextrin (Figure S2C) is the presence of a peak at approximately 1750 cm^−1^ as the characteristic of the ester group related to PCL. This signal is typical of the PCL spectra (Figure S2D, approximately 1760 cm^−1^). The intensity of hydroxyl stretching groups in dextrin (3400 cm^–1^) was slightly decreased in the spectrum of the grafted copolymer with a high dextrin ratio (Figure S2B). The characteristic peaks at 1750 and 3400 cm^–1^ in spectra A and B, respectively, illustrate that dextrin has initiated the polymerization of ε-CL.

Thermal analysis of the dextrin-g-PCL copolymers was carried out by differential scanning calorimetry. The selected representative thermograms are shown in Figures S3 and S4 (Supporting information). Homopolymeric polycaprolactone ensures a melting temperature T_m_ of approximately 72 °C, whereas the melting endotherm T_m_ of the dextrin-g-PCL copolymer tends to be in the range of 59–64 °C. This displacement is due to the varying molar mass of PCL chains grafted to dextrin [30]. The heat of fusion is dependent on the ratio of grafting, and it increases with the number of caproyl units in the copolymer. On the thermograms of the copolymers, the glass transition temperature was determined according to the presence of the grafted caproyl chains, and it is similar (from −58 °C to −62 °C) to that of the caprolactone homopolymer (−61 °C). On the thermograms of these copolymers, the glass transition temperature associated with the dextrin chains was not observed. The thermograms thus confirmed the presence of caproyl chains, which was due to changes in the properties of the material. The obtained copolymers were practically insoluble in water and showed strong swelling in chloroform.

### 3.2. Properties of PLAGA-Block-PEG/Dextrin-Graft-PCL and PLAGA-Block-PEG/Dextrin Blends

The obtained graft-copolymers were used to form polymer blends. A series of blend samples were prepared with dextrin-grafted copolymers with varying degrees of polycaprolactone substitution. The second component of the produced blends was a terpolymer of lactide, glycolide, and PEG. Based on our earlier studies, the terpolymer was selected as an especially useful, good material to formulate biodegradable matrices for application in the release of biologically active compounds to the soil [23]. In the present study, copolymers of dextrin with Rd = 70 wt % and 31 wt % and maltodextrin of Rd = 38 wt % and 20 wt % were selected for application. The blends were obtained by co-dissolution of grafted dextrin with the PLAGA-*block*-PEG terpolymer and with a mass ratio of 50:50 and 30:70. Blends containing more than 50 wt % of modified dextrin showed insufficient mechanical properties, and using them was very difficult to obtain a proper film. The obtained PLAGA-*block*-PEG/dextrin-*graft*-PCL blends were prepared as thin films. The properties of the blends thus prepared were compared with those containing unmodified dextrin or maltodextrin. The composition of the formed blends and basic properties are presented in Table 2.

**Figure 2:**
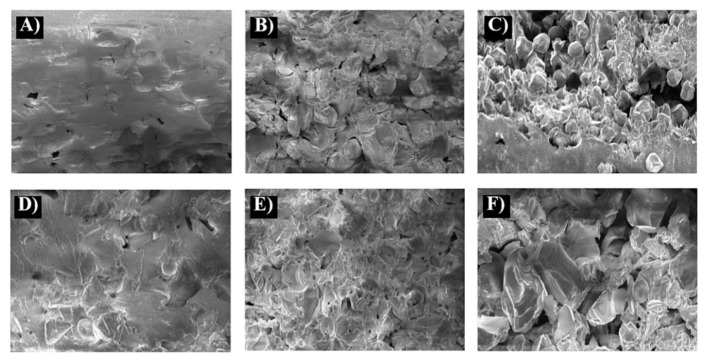
SEM images of blends (after cross section), magnification 500 μm (**A**) sample 2D (LA/GA/PEG D-g-PCL50:50; R_dex_ = 70 wt %); (**B**) sample 11D (LA/GA/PEG + (D-g-PCL); 50:50 R_dex_ = 31 wt %); (**C**) sample OD2 (LA/GA/PEG + D. unmodified) 50:50; and (**D**) sample 2M (LA/GA/PEG + M-g-PCL; R_dex_ = 38 wt %) 50:50; (**E**) sample 11M (LA/GA/PEG + M-g-PCL; R_dex_ = 20 wt %) 50:50; and (**F**) sample OM2 (LA/GA/PEG + M. unmodified) 50:50.

Figure 2 shows representative scanning electron microscopy (SEM) images of morphology of the formed blends. It was observed that when we used the composition 11D containing 50 wt % dextrin-*graft*-polycaprolactone with R_dex_ = 70 wt %, the sample of the formed blend was virtually homogenous (Figure 2A). For blends containing dextrin modified with a larger amount of polycaprolactone (R_dex_ = 31%), phase incompatibility was formed, manifested by the presence of significant gaps between the PLAG-*block*-PEG matrix and modified dextrin grains (Figure 2B). This observed phenomenon is probably related to differences in the length of the caproyl blocks and their number. In the example of a composition formed with unmodified dextrin, the resulting blend appears as immiscible, heterogeneous with a clearly visible two-phase structure containing spherical microparticles of dextrin (Figure 2C). The blends obtained in compositions with modified maltodextrin, even with a high content of polycaprolactone (R_dex_ = 20 wt %) are substantially homogeneous (Figure 2D,E). In the blend where the unmodified maltodextrin was used during molding, this polysaccharide forms a distinct separate typical phase [31], consisting of individual particles of different sizes and shapes and not interlinked with each other (Figure 2F). The lack of modification of dextrin or maltodextrin causes the formation of the immiscible blends.

**Figure 3:**
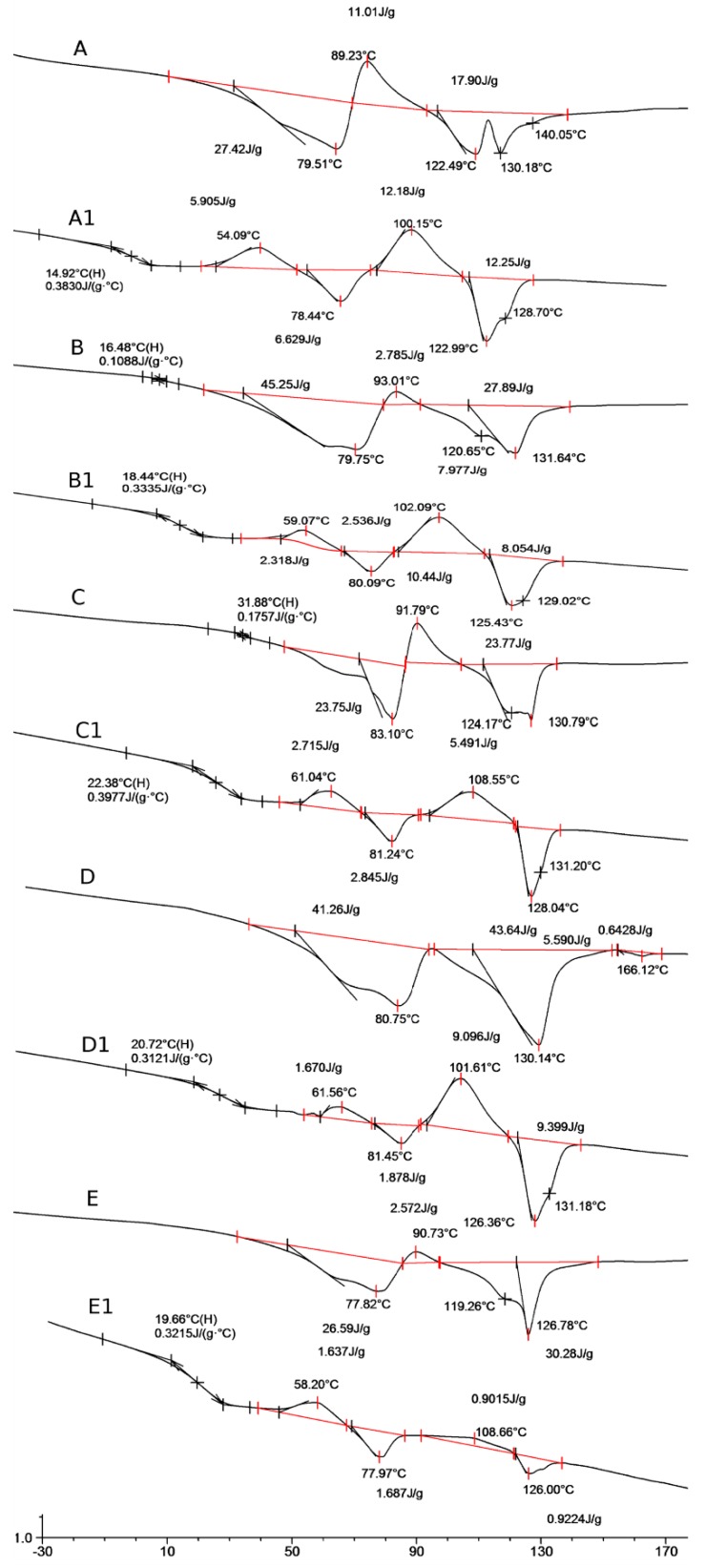
DSC thermograms of the PLGA-block-PEG/dextrin-g-CL blends, (**A**) 1D run I, (**A1**) 1D runII; (**B**) 2D run I, (**B1**) 2D run II; (**C**) 10D run I, (**C1**) 10D run II; (**D**)11D run1, (**D1**) 11D run II; (**E**) OD2 run I, (**E1**) OD2 run II.

The obtained DSC thermograms of the blends (Figure 3 and Supporting Information, Figure S5) were substantially similar to the previously published thermograms of PLAGA-*block*-PEG terpolymers [23]. However, the blends present a slightly lower glass transition temperature, which depends on the composition and ranged from 15 °C to 22 °C for blends of grafted dextrin and from 20 °C to 24 °C for blends containing grafted maltodextrin. Because of the presence of enthalpy of crystal phases and strong relaxation enthalpy on the presented thermograms of dextrin’s blends and copolymer PLAGA/PEG, it is quite difficult to observe and discuss the thermal effects at temperatures above 40 °C where the glass transition temperature of dextrin may occur. High temperature used during DSC measurements and heating amorphous samples to 180 °C and then rapid cooling showed no enthalpy on obtained thermograms (Supported Information, Figure S6).

On the basis of these DSC thermograms, only one glass transition temperature was observed for all the blends; although this confirms earlier conclusions about their miscibility, it should be noted that the glass transition temperature can be very difficult to observe for many dextrin and maltodextrins. All obtained thermograms demonstrated endotherms, which were assigned to the ordered lactidyl blocks of the PLAGA/PEG copolymer. This phase was melted at temperatures ranging from 122 to 133 °C. Because of the presence of relaxation endotherm and based on the first run of DSC, it was not possible to determine the melting point related to the ordering of caproyl blocks or PEG. Thermograms obtained after glass transition of samples (run II) exhibited beside the crystallization and melting enthalpy of ordered lactidyl blocks, occurring of small additional crystallization exotherm ca 60 °C and corresponding it melting endotherm at temperature about 80 °C (3–6 kJ/g) which probably corresponds to presence of ordered caproyl domens grafted on dextrin in these blends. However, due to a large width and shape of this endotherm, we cannot exclude the contribution of domains originating from the ordering of PEG blocks. Practically, the exotherm for terpolymers PLAGA-block-PEG occur in the same temperature range of 70–85 °C [23]. Thermograms of blends with unmodified dextrin show only one essential crystallization and melting zone assigned to lactydyl blocks. In this blend, the content of ordered PEG block domains is too small to observe the essential enthalpy that could confirm their presence. To explain the appearance of endotherms of blends containing grafted maltodextrin, additional measurements were performed in run III condition. The measurement was conducted at a relatively slow heating speed of 10 °C/min on the recrystallized samples by reheating and slow cooling. The obtained thermograms are shown in Figure S5A2,B2,C2 (Supported Information).

Indeed, all thermograms of the already presented blends containing grafted dextrin demonstrate the endotherms of crystallization and melting phases, which correspond to the ordering of PEG and caproyl (two signals of crystallization exotherm at temperature ca 52–55 °C and melting endotherm at ca 80 °C) as well as lactidyl blocks (strong crystallization endotherms ranging in the temperature of 90–100 °C, and melting exotherms in the temperature range between 122 °C and 132 °C). Blends containing nongrafted maltodextrin demonstrate only the presence of one crystalline phase assigned to lactidyl microblocks. Overall, the results of DSC confirmed the conclusions obtained from morphological observations of blends (based on SEM photographs).

### 3.3. Biodegradation Process of Blends

**Figure 4:**
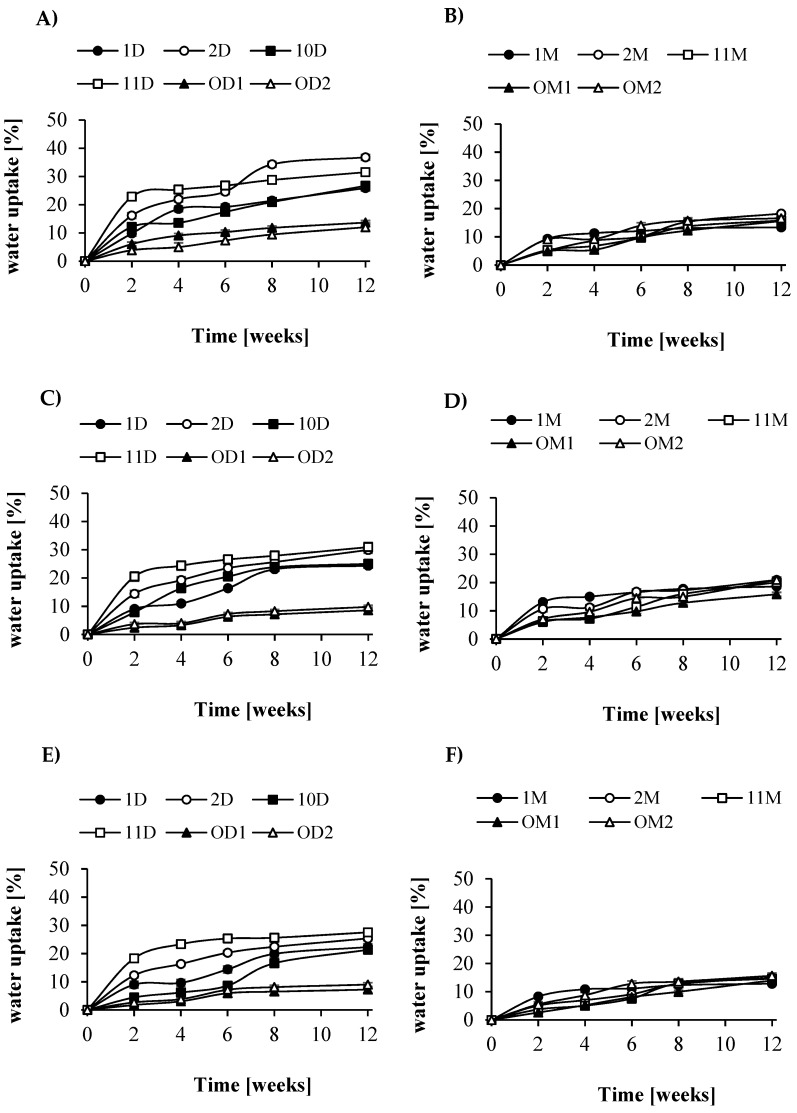
Water uptake of the samples: (**A**)—LA/GA/PEG + dextrin in water, and (**B**)—LA/GA/PEG + maltodextrin in water; (**C**)—LA/GA/PEG + dextrin in soil, and (**D**)—LA/GA/PEG + maltodextrin in soil; (**E**)—LA/GA/PEG + dextrin in activated sludge and (**F**)—LA/GA/PEG + maltodextrin in activated sludge.

**Figure 5:**
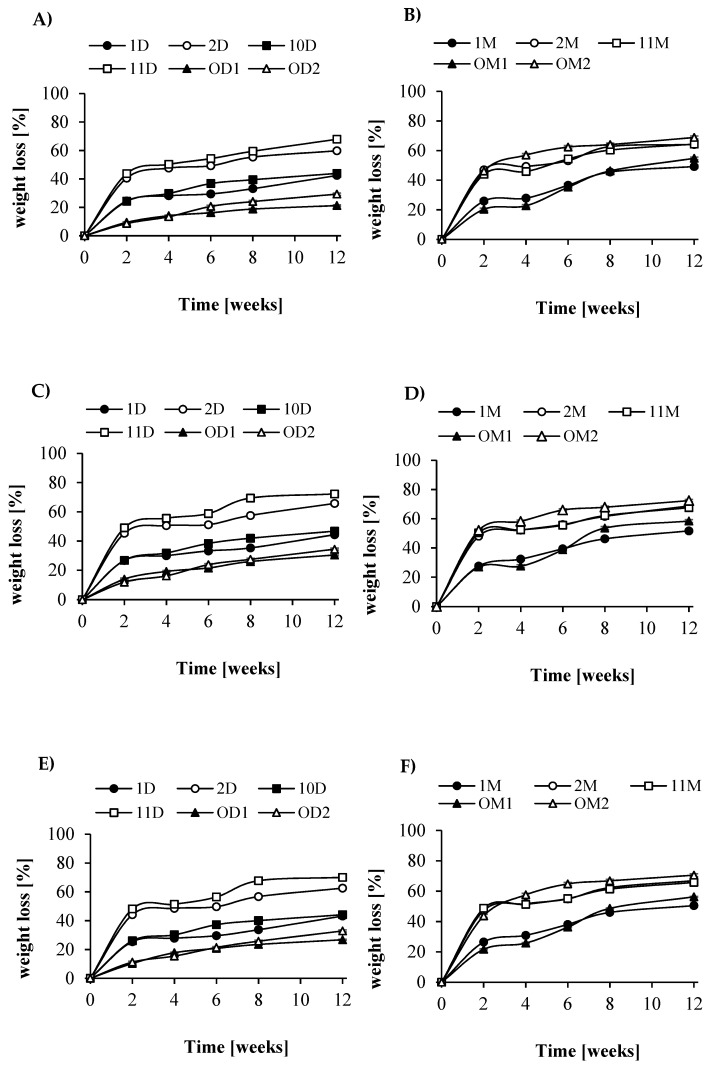
Percent weight loss of the samples: (**A)**—LA/GA/PEG + dextrin in water, and (**B)**—LA/GA/PEG + maltodextrin in water; (**C**)—LA/GA/PEG + dextrin in soil, and (**D**)—LA/GA/PEG + maltodextrin in soil; (**E**)—LA/GA/PEG + dextrin in activated sludge and (**F**)—LA/GA/PEG + maltodextrin in activated sludge.

**Figure 6:**
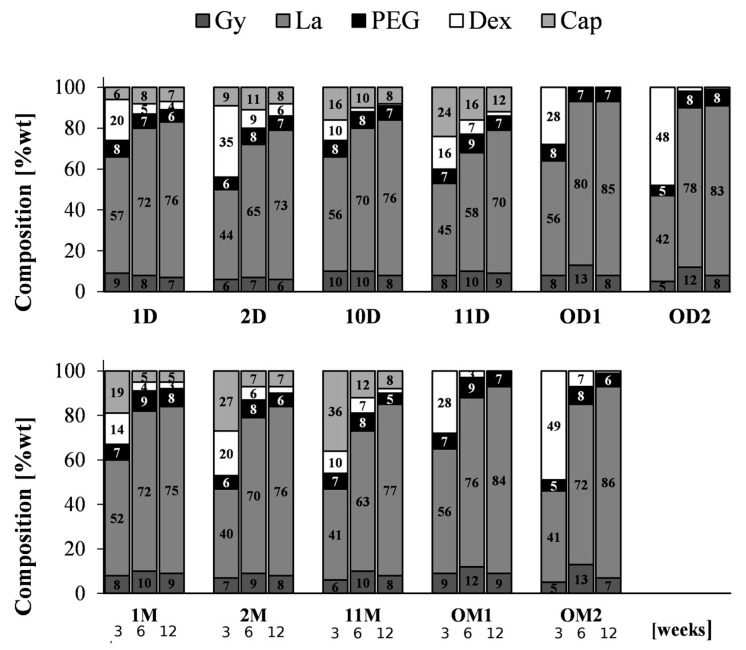
Changes of the blends composition during degradation process conducted in soil (wt.%) (estimated based on 1H NMR measurement).

In order to understand the effect and significance of the biodegradation course of the obtained mixtures on the herbicide release profile, the degradation experiments were conducted in water, soil, and activated sludge. Soil (S) is a target environment characterized by a minimum amount of water and the presence of microorganisms. In water (W) the mechanism of degradation by hydrolysis of ester bonds is tested. In activated sludge (AS), the degradation mechanism is observed by both hydrolysis and enzyme activity produced by microorganisms. An increase in water absorption (Figure 4) and a decrease in weight loss (Figure 5) were recorded. Based on the NMR signals of protons (Supporting Information, Figures S7 and S8, Equations (S1)–(S5)) associated with lactidyl CH (5.2 ppm), glycolidyl CH_2_ (4.75–4.95 ppm), caproyl CH_2_–O–(3.9–4.25 ppm) units, CH_2_ groups of PEG (3.5 ppm), and anomeric hydrogen saccharide protons (5.4–5.6 ppm), the blend composition and changes occurring during soil degradation were estimated. The results are summarized in Figure 6. The changes in blend sample composition were strongly correlated with changes in their weight loss observed during the degradation time (Figure 5). It was found that grafted dextrin or maltodextrin in tested blends was mostly degraded after 6 weeks of the experiment. Rapid weight loss of samples at the initial phase of the degradation process was due to the release of degradation products into the medium. Hence, the highest weight loss was noted for blends containing by weight 50% of grafted dextrin (samples 2D and 11D) or maltodextrin (2M and 11M) containing the highest amount of saccharide. After 6 weeks of incubation of samples in soil, only few percent weight loss of grafted dextrin and maltodextrin was observed. A decrease in grafted caproyl sequences was observed; however, this decrease was much lower than that for the dextrin one. For 2D blend, the initial mass ratio of dextrin to caproyl was approximately 3.9, and after 6 weeks and 3 months, it was 0.82 and 0.75, respectively. A similar phenomenon was found for all blends containing grafted dextrin and caproyl side chains with relatively long average length (100–150 caproyl units). A slight difference in degradation characteristic was observed for blends with grafted maltodextrin. For instance, the initial ratio by weight of maltodextrin to caproyl of blend 2M was 0.75, which was 0.6 and 0.43, respectively, after 6 weeks and 3 months. After 6 weeks of incubation, all tested blends had a similar composition (Figure 6), and as a consequence, further degradation steps were similar for all samples. As predicted, the amount of water absorption was basically related to the degradation environment (Figure 4). Samples that were immersed in water, as degradation and surface erosion progressed, were characterized by a gradual increase in water uptake. The highest water uptake (approximately 37% by weight in water) was obtained in samples of films formed from 2D blends containing grafted dextrin in the ratio 50:50. Significant differences were observed in water uptake between types of samples depending on their wettability. The lowest absorbency was found in samples with the lowest wettability and in samples of films made of blends in the proportions of 50:50 and 70:30 in all media.

Similar results were obtained for films formed from blends (PLAGA-*block*-PEG)-*blend*-(maltodextrin-*graft*-PCL); however, the water uptake was much lower than that for blends where dextrin was used (approximately 10%). The observed lower water absorption of these blends is associated with a higher content of hydrophobic caproyl units. Figure 5 presents the weight loss of the test samples during incubation in different media. As already mentioned, observed rapid weight loss of samples at the initial phase of degradation (2–3 weeks) corresponded to rapid degradation of grafted dextrin or maltodextrin. After this time, the rate of weight loss was much lower. After 12 weeks of degradation, the largest decrease in the mass of the active sludge (of approximately 60–70%) was observed for blends composed of LA/GA/PEG and D-g-PCL; R_dex_ 70 wt % in ratio 50:50 (sample 2D) and for LA/GA/PEG and D-g-PCL; R_dex_ 31 wt % in ratio 50:50 (sample 11D). These results are consistent with the water absorption characteristics and their wettability.

Unexpectedly, it was found that the degradation rate of tested blends was mostly dependent on the content of lactidyl blocks but not on grafted dextrin. Samples containing a higher amount of lactidyl (1D and 10D with approximately 56 wt % lactidyl units) degraded significantly slower than samples 2D and 11D (approximately 41–45 wt % lactidyl units). After 12 weeks, the weight loss of samples reached only ca 40%. Interestingly, the degradation rate in AS was very similar to the degradation process in soil. Here, the largest weight loss was noted for samples 11D and 2D and reached ca 60–70%. Similar results were also observed for degradation in water. Independent of the medium used, the lowest degradation value was found for blends containing unmodified dextrin (OD1 and OD2). It is worth noting that the decrease in weight loss for samples containing maltodextrin was generally analogous to the previous one. In the AS, the weight loss was slight but noticeable—approximately 60%. However, the largest level of degradation (over 70%) was recorded during the degradation process conducted in soil. The blends with nongrafted maltodextrin degraded in almost the same way as the corresponding blends with a similar composition containing modified maltodextrin. This is due to the good solubility of maltodextrin in water and the effect of leaching of this compound. From the degradation process of the polymeric samples in water, soil, and AS, their ongoing surface erosion was measured by SEM.

The most significant changes in surface erosion of the polymeric samples after 12 weeks of degradation are illustrated in Supporting Information, Figure S9.

To compare surface erosion, the samples were collected after 3 months of incubation in water, soil, and AS. The morphological changes of the polymer surface depended on the composition and incubation medium. An analysis of microscopic images showed that the largest changes in the morphology occurred for those samples that were incubated in the active sludge environment, and slightly less level of erosion was noted for samples degraded in soil. This effect is particularly evident for blend samples containing 50% modified dextrin with R_dex_ = 31 wt % (sample 11D, Figure S9). This indicates that these samples were most subjected to enzymatic degradation. Similar blends containing nonmodified dextrin also showed strong changes, but mainly due to the gradual leaching of this polysaccharide as shown by fairly regular cavities arising after deletion of dextrin grains (sample OD2, Figure S9)

### 3.4. Release of Selected Herbicides

The release rate of immobilized herbicides was dependent on their solubility in water, composition of polymeric blends, and amount of herbicides loaded into polymers. For agricultural applications, the release rate of active substances should be optimal in the growing vegetation of soil. The optimal dose of releasing herbicide should be reasonably constant throughout the growing season, and its residue should be minimal after this period. Therefore, it is necessary to design a polymeric matrix that can release pesticides in optimal doses, effective against undesirable weeds. Initially, the high rate of herbicide release is caused by direct elution of the active substances from the polymer surface; later it results from diffusion through the micropores and the rate of matrix degradation in a hydrolytic or enzymatic process. In our study, herbicides used in soil metazachlor and pendimethalin were chosen as model compounds with high practical significance and significant differences in water solubility.

In an earlier study [23], it was shown that the hydrophobicity of the carrier affects the release level of the herbicide. Where the used matrix used is a hydrophobic polymer such as PLGA copolymer, the observed release rate of metazachlor and pendimethalin is slow, with a total amount of released active ingredient less than 3% and 5% respectively of the initial value. Rapid leaching of water-soluble PEGs into the soil during degradation facilitated the release of herbicide immobilized in the polymer matrix, as evidenced by samples without the addition of grafted dextrin/maltodextrin.

In the present experiment, the burst effect for all tested herbicides took approximately only 1 week. The burst release mechanism depends on the nature of the active substance delivery system. Rapid release may be due to the high specific surface that is in contact with external media; in this case, it was AS, soil, and water. The reason why burst release occurs maybe because immobilized polymer herbicides prepared through the solvent casting method can be scattered over or be localized under the surface of the carrier, and when they come in contact with the medium, it causes a high burst release.

**Figure 7:**
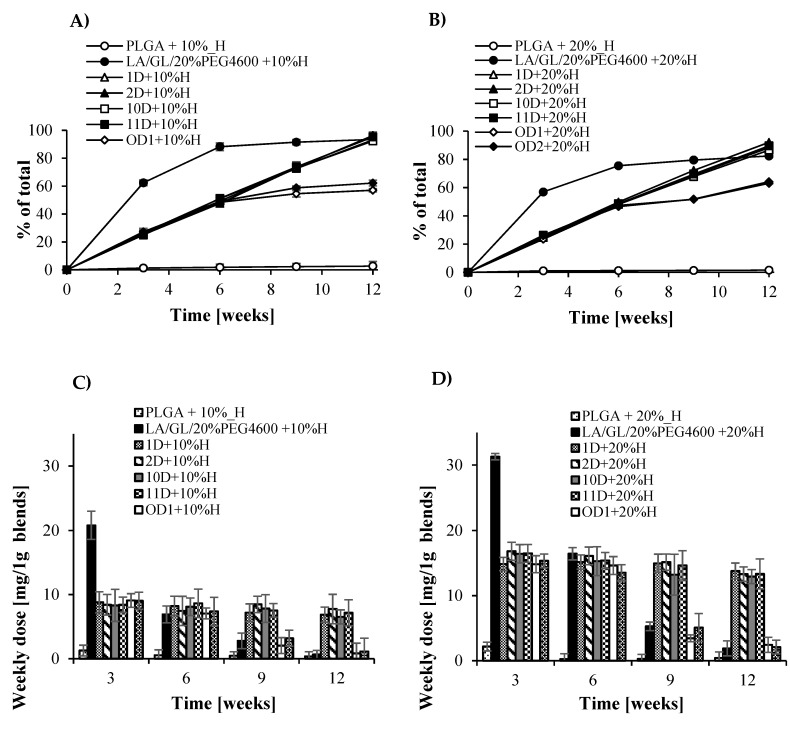
Cumulative (**A**,**B**) and weekly dose release (**C**,**D**) of metazachlor in soil (dextrin).

Because metazachlor has relatively good solubility in water, for blend containing copolymer PLAGA/PEG and dextrin-*graft*-PCL or maltodextrin-*graft*-PCL, a tendency of higher release of metazachlor in both soil and water was observed for the first 3 weeks. The burst effect was the most noticeable for carriers formed with blends containing modified oligosaccharides. The rate of metazachlor release for this system was directly influenced by the amount of herbicide loaded into polymer carriers. Comparing the burst effect of metazachlor released to water and soil, it is worth noting that in water this effect was much stronger than in soil. This aspect is promising for agrochemical applications because it will reduce the release of excessive amount of herbicide into the soil. Blends containing grafting of dextrin and maltodextrin with 10% metazachlor showed almost 100% release to the soil within 12 weeks, while blends containing 20% of metazachlor showed almost 90% release (Figure 7 and Supporting Information, Figures S10–S12).

The release rate of pendimethalin from all blends in water was much lower than that of the abovementioned herbicide and equaled approximately 30–40% and 25–45%, respectively, for blends containing 10% and 20% herbicide. The total percentage release of pendimethalin into the soil within 12 weeks was less than 55% of its total quantity (Supporting Information, Figures S13–S16).

A comparison of the cumulative release of metazachlor and pendimethalin showed that the plateau phase was not reached for the latter herbicide. The release of pendimethalin was still in the growth phase, even after 12 weeks. Average weekly released doses of this herbicides, over the observed time, were almost the same. The principal factor contributing for the reduced release rate of pendimethalin is its solubility in water. The solubility of metazachlor in water is 450 mg/L, which is more than a thousand times greater than the solubility of pendimethalin, which is 0.33 mg/L. For the blend prepared with oligosaccharides unmodified for both tested herbicides, the release was significantly lower in all cases. For metazachlor, the release values were lower by approximately 20% and by 30% for pendimethalin). Modification of the analyzed saccharides affects the improvement of the release profile, which is particularly noticeable during the release of the herbicide in the soil. The weekly release rates for both metazachlor and pendimethalin were constant for a period of 12 weeks.

Comparison of the NMR and UV–Vis spectra of the used herbicides before and after the release experiment proved that the morphology of these substances did not change during the immobilization time and after the release experiment (Supporting Information, Figure S17). This means that the proposed controlled release system protects the used herbicides against their degradation caused by environmental conditions such as moisture, UV radiation, microbial activity, or temperature and allows for their release as unchanged substances even after few months of immobilization.

## 4. Conclusions

Among all the tested blends, which were proposed as a carrier for potential use in controlled release of plant protection agents, PLGA-block-PEG copolymer blended with grafted dextrin yielded very promising results for their future applications. In the 3-month release experiment, almost 90% of the loaded herbicides were gradually released into the soil; this is very important from the agricultural point of view, because the average growing season of cultivated plants is approximately few months (Figure 7). The optimal time of herbicide release to the root is most of the vegetation time of the plant (for cereals it is from 3 to 4 months). Considering the high susceptibility of modified dextrin on enzymatic action in soil, its presence in blend essentially accelerated the first phase of sample degradation when compared to the copolymer PLAGA/PEG. Enzymatic degradation of modified dextrin combined with leaching of the degradation products into medium caused significant erosion of the polymer matrix, thereby leading to acceleration of water diffusion into the polymer matrix and allowing for easier leaching of herbicides outside the matrix. This phenomenon reflects the gradual release process of metazachlor in soil as compared to its release in water. As already mentioned, the proposed formulations provide herbicides in an unchanged form into soil within a few months of release. Among the tested formulations with immobilized metazachlor, those containing dextrin were much more effective than maltodextrin carriers, probably due to differences in the water absorption characteristics of the used materials. The tested controlled release system is not effective in the presence of the herbicide pendimethalin probably due to its extremely low solubility in water, which is one of the most important factors determining the release rate of immobilized active substances. Although pendimethalin immobilized in the blend containing modified dextrin exhibited a gradual release process within 3 months, it was not satisfactory because only 40% of the total loaded amount of herbicide was released.

## Figures and Tables

**Table 1:** Conditions of the grafting reactions and characteristic of the final copolymers

No. Sample	Time of Reaction (h)	Weight Ratio Dextrin/CL in Reaction Mixture	Caprolactone Conversion (wt %)	Average Chain Length of Polycaprolactone (Cap units)	R_dex_ Weight Ratio Dextrin/PCL in the Copolymer (wt %)
**1**	48	50:50	96	231	75
2	**48**	**30:70**	**94**	**152**	**70**
**3**	72	25:75	97	140	58
**4**	48	15:85	93	104	46
5	**72**	**10:90**	**96**	**96**	**31**
		**Weight Ratio Maltodextrin/CL in Reaction Mixture**			**R_dex_** **Weight Ratio Maltodextrin/PCL in Copolymer (wt %)**
6	**48**	**30:70**	**97**	**96**	**38**
7	48	25:75	96	97	23
8	48	15:85	96	98	26
9	**72**	**10:90**	**97**	**94**	**20**

The bolded font indicates the copolymers that were selected as a component of the formed blends.

**Table 2:** Composition and thermal properties of polymer blends used in the study

No	Blend Composition	Weight Ratio of the Blend Components	Weight Ratio of LA:GL:PEG: DEX:CAP Units	T_g_ (°C)	T_m_ (°C)	ΔH (J/g)
**0**	LA/GA/PEG	100	78:12:10:0:0	37	82 125	3 19
**1D**	(LA/GA/PEG) + (Dextrin-g-PCL; R_dex_ = 70 wt %)	70:30	55:8:7:21:9	15	78 123	7 12
**2D**	(LA/GA/PEG) + (Dextrine-g-PCL; R_dex_ = 70 wt %)	50:50	40:5:5:36:13	18	80 125	3 8
**10D**	(LA/GA/PEG) + (Dextrine-g-PCL; R_dex_ = 31 wt %)	70:30	55:8:7:9:21	22	81 128	3 6
**11D**	(LA/GA/PEG) + (Dextrine-g-PCL; R_dex_ = 31 wt %)	50:50	40:5:5:15:35	21	81 126	2 9
**OD1**	(LA/GA/PEG) + Dextrine.UNMODIFIED	70:30	55:8:7:30:0	21	130	1
**OD2**	(LA/GA/PEG) + Dextrine.UNMODIFIED	50:50	40:5:5:50:0	20	127	1
**1M**	(LA/GA/PEG) + (Maltodextrine-g-PCL; R_dex_ = 38 wt %)	70:30	55:8:7:11:19	21	81 125	6 19
**2M**	(LA/GA/PEG) + (Maltodextrine-g-PCL; R_dex_ = 38 wt %)	50:50	40:6:5:19:30	20	80 122–130	4 11
**11M**	(LA/GA/PEG) + (Maltodextrine-g-PCL; R_dex_ = 20 wt %)	50:50	40:6:5:10:39	24	80 124–131	4 11
**OM1**	(LA/GA/PEG) + Maltodextrine.UNMODIFIED	70:30	55:8:7:30:0	25	127–132	7
**OM2**	(LA/GA/PEG) + Maltodextrine.UNMODIFIED	50:50	40:6:4:50:0	25	125–132	7

T_g_—glass transition; T_m_—temperature of melting crystalline phase; ΔH—endotherm of crystalline phase melting. Weight ratio of monomers units in blends was calculated theoretically based on the weight ratio of the used components.

## Supplementary Materials

The following are available online at https://www.mdpi.com/1996-1944/13/4/832/s1, Figure S1: Calibration curve of herbicides solution standards, Figure S2: FTIR spectra of homopolymers and the graft copolymer; (A)—dextrin-g-PCL (dextrin contain—31wt.%); (B)—dextrin-g-PCL (dextrin contain —70 wt.%); (C)—dextrin from corn; (D)—PCL, Figure S3: DSC thermograms of the graft copolymer dextrin-g-PCL, Figure S4: DSC thermograms of the graft copolymer maltodextrin-g-PCL, Figure S5: DSC thermograms of the PLGA-block-PEG/maltodextrin-g-CL blends, (A) 1M run I, (A1) 1M run II, (A2) 1M run III, (B) 2M run I, (B1) 2M run II, (B2) 2 M run III, (C) OM2 run I, (C1) OM2 run II, (C2) OM2 run III, Figure S6: Thermogram sample 1D, heating 20 °C/min, IV-run, T_g_ = 15 °C, Figure S7: 1H NMR spectra of 1D blend (DMSO D6); (A) before degradation, (B) after 6 weeks of incubation in soil, (C) after 12 weeks of incubation in soil, Figure S8: 1H NMR spectra of 1M blend (DMSO D6); (A) before degradation, (B) after 6 weeks of incubation in soil, (C) after 12 weeks of incubation in soil, Figure S9: SEM images of the degradation (after cross section, magnification 500 μm) 2D (LA/GA/PEG+D-g-PCL; R_dex_ = 70 wt.%) 50:50; 11D (LA/GA/PEG+D-g-PCL; R_dex_ = 31 wt.%) 50:50 and OD2 (LA/GA/PEG + D.UNMODIFIED 50:50 (100 μm), Figure S10: Cumulative (A,B) and weekly dose release (C,D) of Metazachlor in water (maltodextrin), Figure S11: Cumulative (A,B) and weekly dose release (C,D) of Metazachlor in water (dextrin), Figure S12: Cumulative (A,B) and weekly dose release (C,D) of Metazachlor in soil (maltodextrin), Figure S13: Cumulative (A,B) and weekly dose release (C,D) of Pendimethalin in water (maltodextrin), Figure S14: Cumulative (A,B) and weekly dose release (C,D) of Pendimethalin in water (dextrin), Figure S15: Cumulative (A,B) and weekly dose release (C,D) of Pendimethalin in soil (maltodextrin), Figure S16: Cumulative (A,B) and weekly dose release (C,D) of Pendimethalin in soil (dextrin), Figure S17: 1H NMR spectra of (A) matrix containing Metazachlor residues after 12 weeks of release, (B) Metazachlor.
