# Supplementary material for: Biodegradable Blends of Grafted Dextrin with PLGA-block-PEG Copolymer as a Carrier for Controlled Release of Herbicides into Soil
Source: Materials (Basel). 2020 Feb 12;13(4):832. doi: 10.3390/ma13040832 (PMC7079626; doi:10.3390/ma13040832)
Supplement: Supplementary file 1 [file materials-13-00832-s001.pdf]

materials

Supplementary Materials

Biodegradable Blends of Grafted Dextrin with PLGA-block-PEG Copolymer as a Carrier for Controlled Release of Herbicides into Soil

Kamila Lewicka [1], Piotr Rychter [1], Małgorzata Pastusiak [2], Henryk Janeczek [2] and Piotr Dobrzynski [1,2,*]

1 Jan Dlugosz University in Czestochowa, Faculty of Science and Technology, 13/15 Armii Krajowej Av., 42-200 Czestochowa, Poland; kamila.lewicka@ujd.edu.pl (K.L.); p.rychter@ujd.edu.pl (P.R.)
2 Centre of Polymer and Carbon Materials, Polish Academy of Sciences, 34 Curie –Sklodowskiej Str., 41-819 Zabrze, Poland; mpastusiak@cmpw-pan.edu.pl (M.P.); hjaneczek@cmpw-pan.edu.pl (H.J.); p.dobrzynski@ujd.edu.pl (P.D.)
* Correspondence: p.dobrzynski@ujd.edu.pl; Tel.: + 48 32 271 60 77

Preparation of herbicides calibration curve

The calibration curve was prepared using a series of samples of each herbicide concentration solutions (herbicide standards in water). A curve was fitted to the data, and the resulting equation was used to convert readings of the unknown samples into concentrations. The unknown concentration of released herbicide was measured mathematically by solving the equation for concentration as a function of signal.

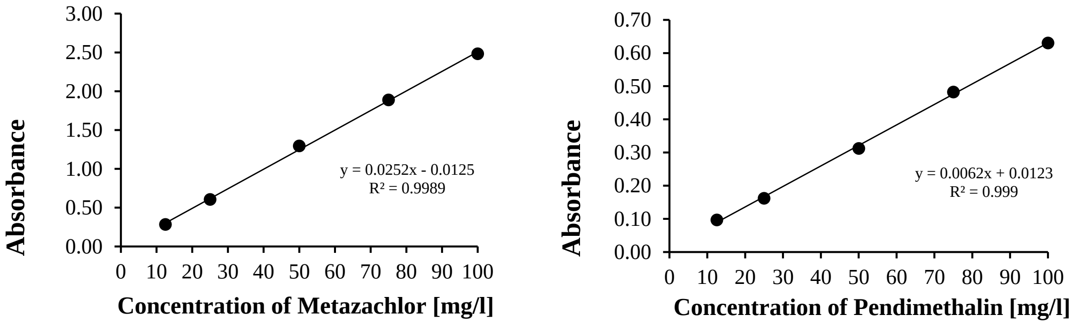

Figure 1S. Calibration curve of herbicides solution standards

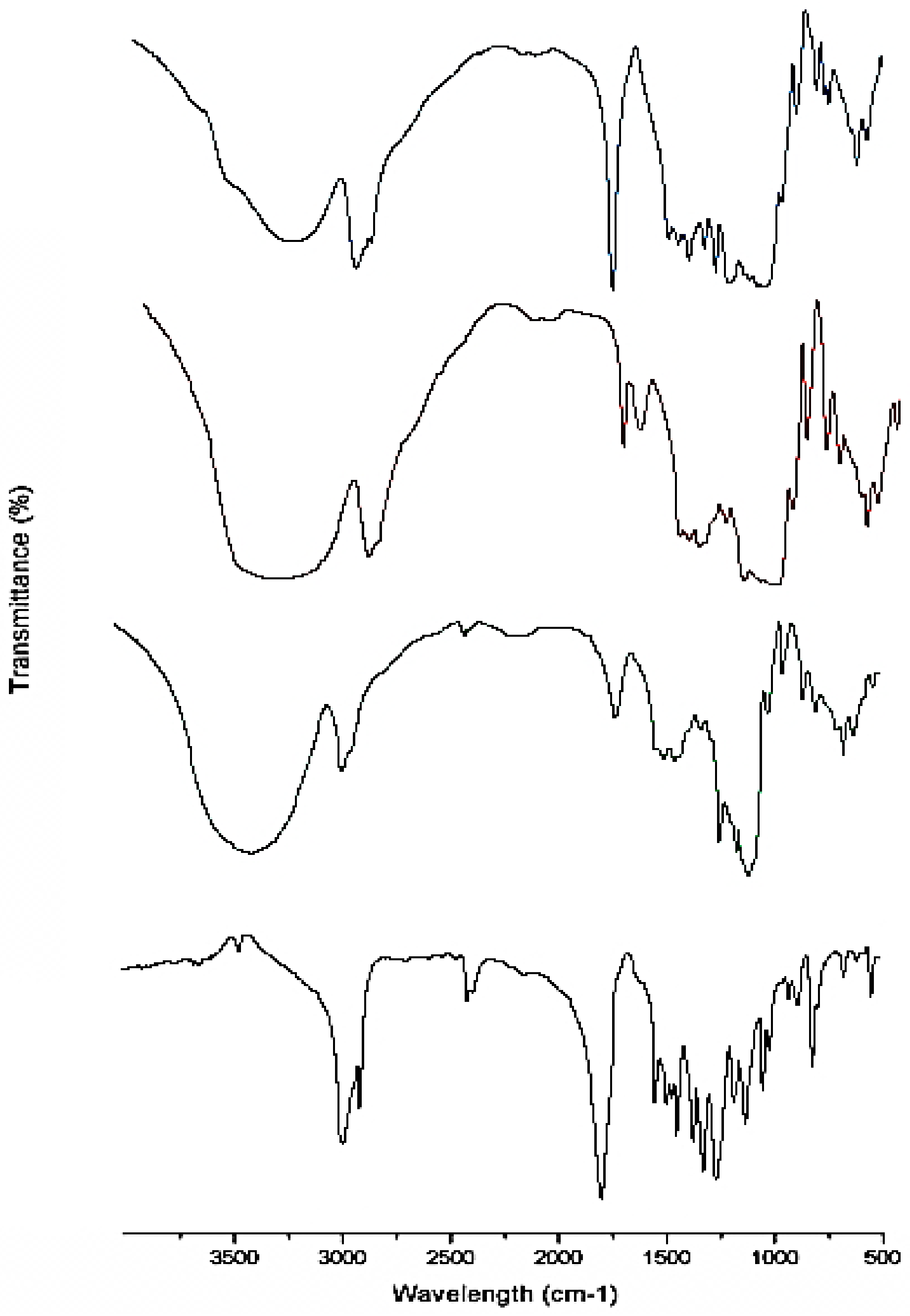

Figure 2S. FTIR spectra of homopolymers and the graft copolymer; (**A**)—dextrin-g-PCL (dextrin contain—31wt.%); (**B**)—dextrin-g-PCL (dextrin contain —70 wt.%); (**C**)—dextrin from corn; (**D**)—PCL.

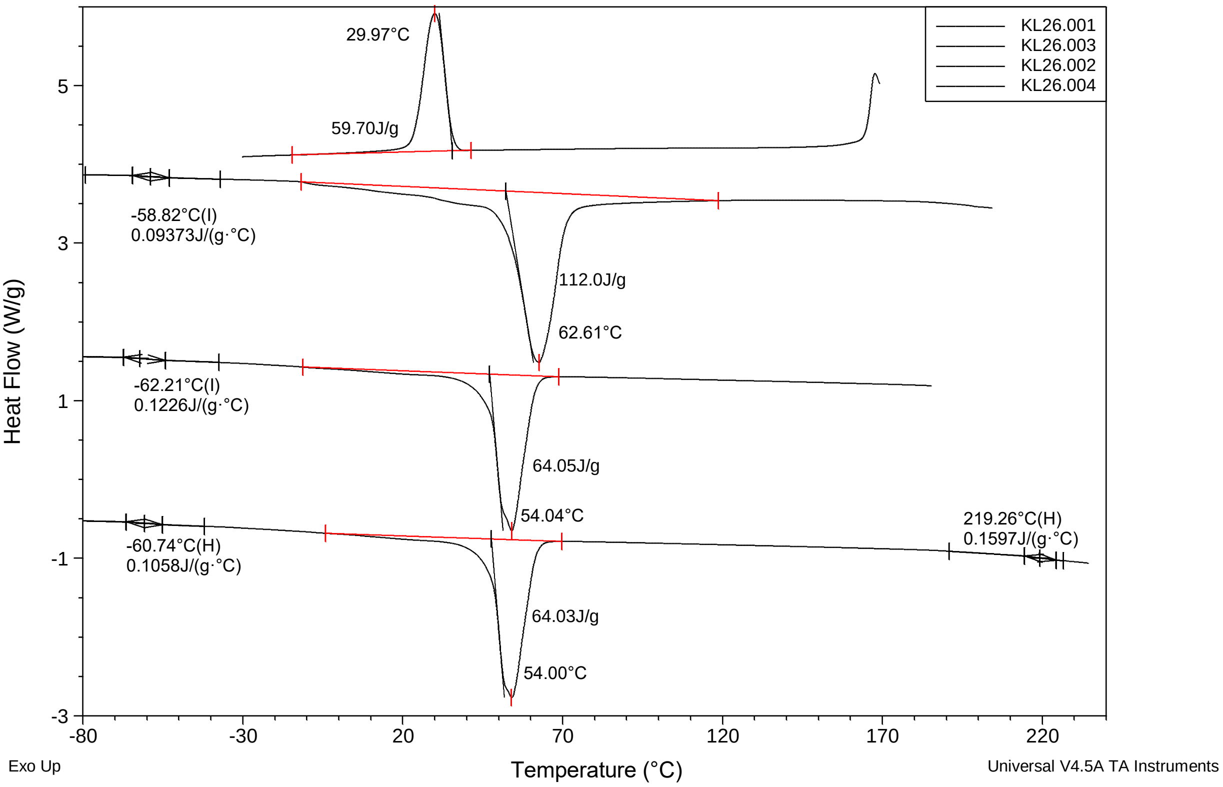

Figure 3S. DSC thermograms of the graft copolymer dextrin-g-PCL.

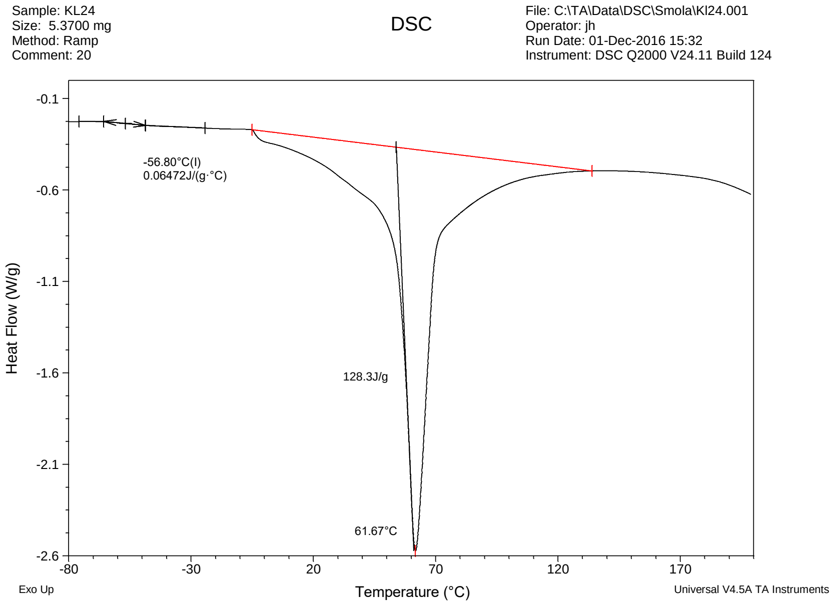

Figure 4S. DSC thermograms of the graft copolymer maltodextrin-g-PCL.

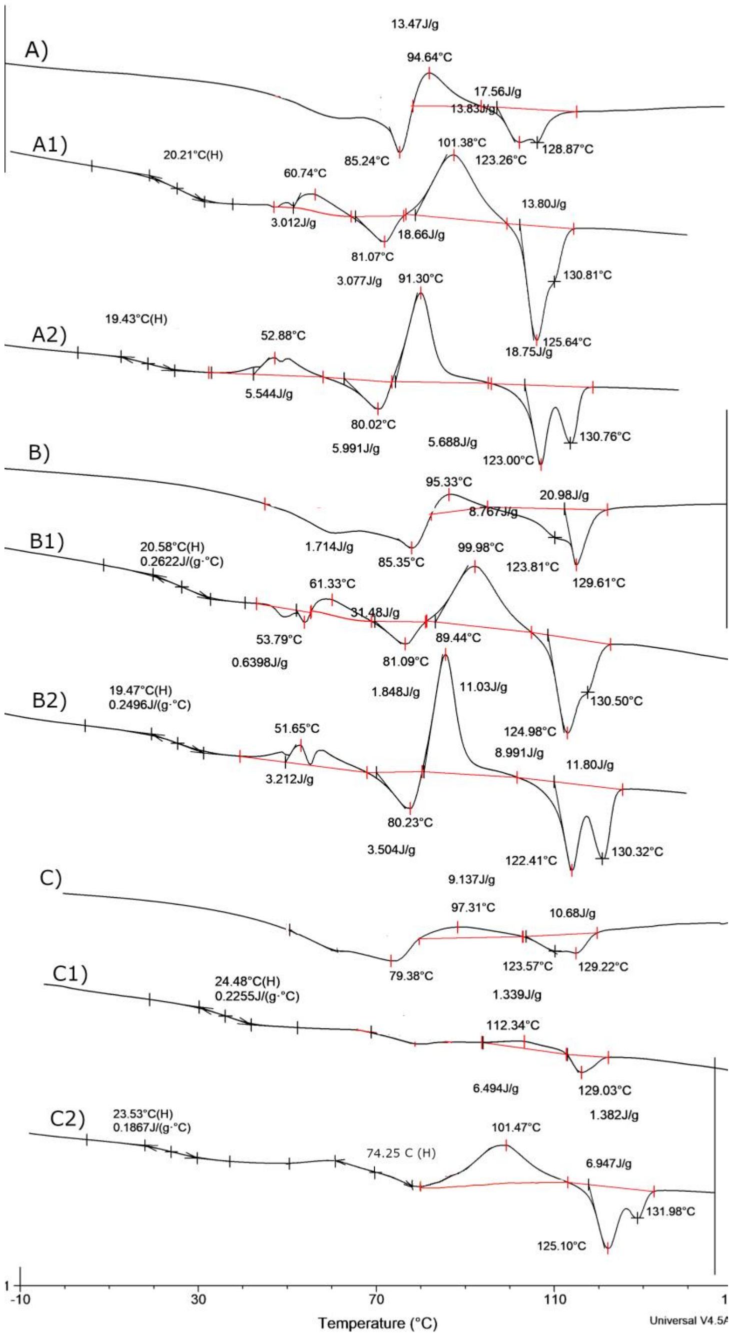

Figure 5S. DSC thermograms of the PLGA-block-PEG/maltodextrin-g-CL blends, (**A**) 1M run I, (**A1**) 1M run II, (**A2**) 1M run III, (**B**) 2M run I, (**B1**) 2M run II, (**B2**) 2 M run III, (**C**) OM2 run I, (**C1**) OM2 run II, (**C2**) OM2 run III.

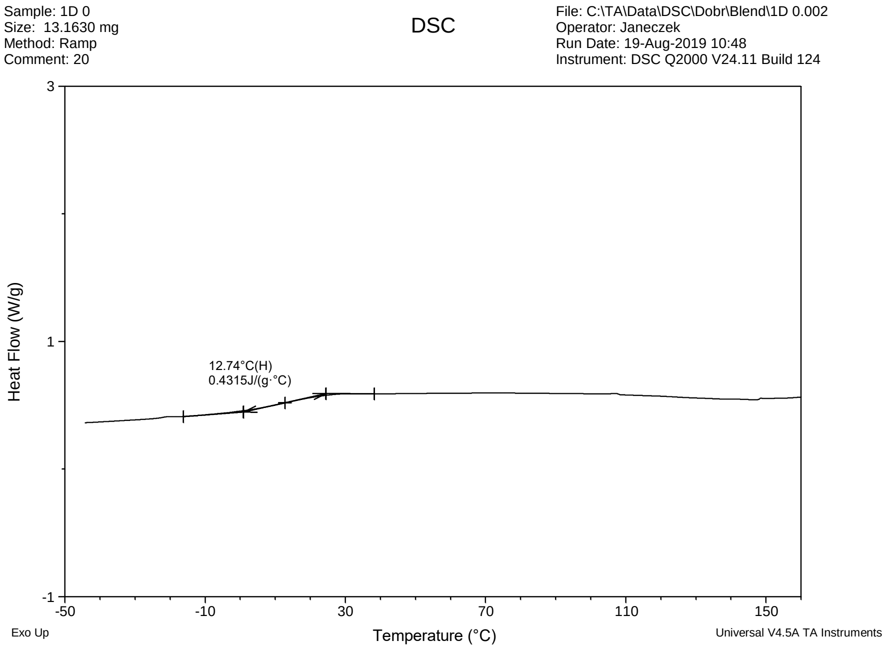

Figure 6S. Thermogram sample 1D, heating 20 °C/min, IV-run, T_g = 15 °C.

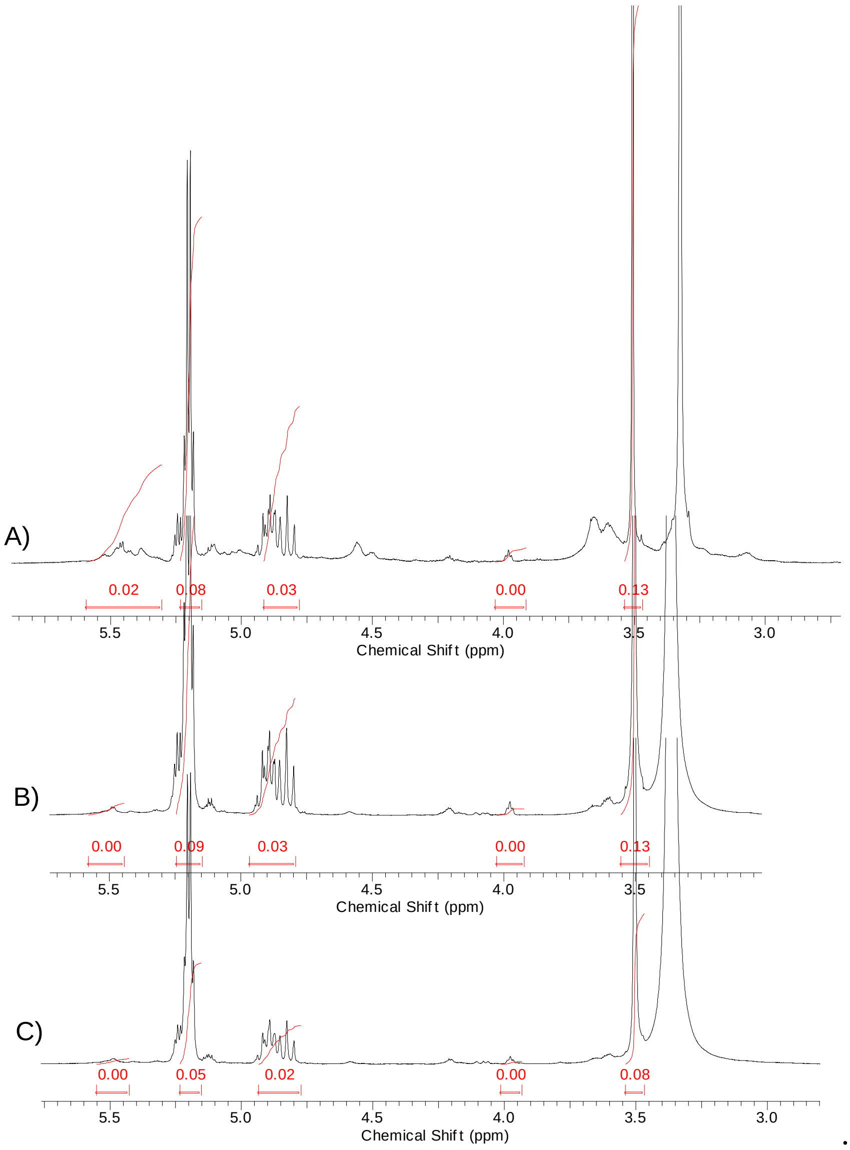

Figure 7S. 1H NMR spectra of 1D blend (DMSO D6); (**A**) before degradation, (**B**) after 6 weeks of incubation in soil, (**C**) after 12 weeks of incubation in soil.

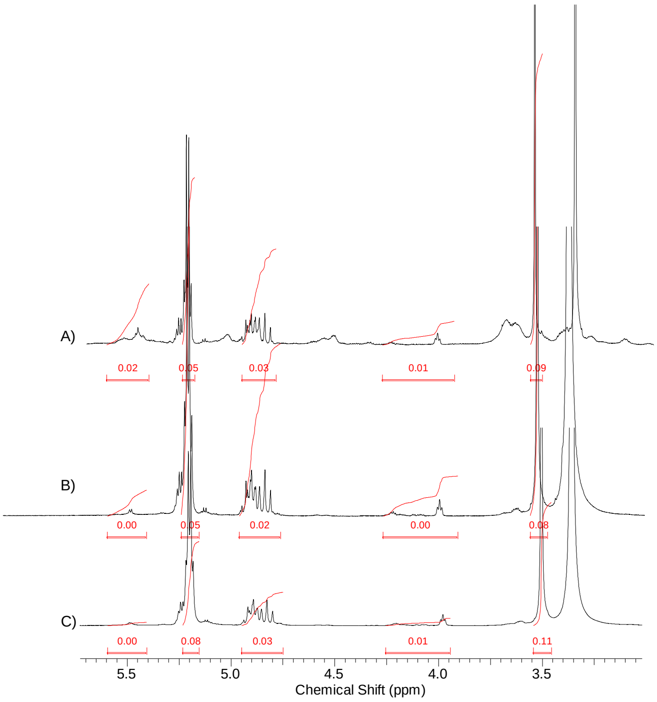

Figure 8S. 1H NMR spectra of 1M blend (DMSO D6); (**A**) before degradation, (**B**) after 6 weeks of incubation in soil, (**C**) after 12 weeks of incubation in soil.

Content (wt. %) of dextrin, lactidyl, glycolidyl, caproyl units and PEG in the blend was calculated according to equations S1, S2, S3, S4 and S5 with 1H NMR spectra;

$$C_{dex}(\text{wt.\%}) = \frac{162 \times I(H1)}{162x\, I(H1)+\frac{114}{2}\, x\, I(\varepsilon)+144\, x\, I(La)+\frac{116}{2}\, x\, I\, (Gl)+\frac{4200}{95.5\, x\, 4}\, x\, I(CH_2)} \times 100\% \quad (S1)$$

$$C_{Cap}(\text{wt.\%}) = \frac{\frac{114}{2}\, x\, I(\varepsilon)}{162x\, I(H1)+\frac{114}{2}\, x\, I(\varepsilon)+144\, x\, I(La)+\frac{116}{2}\, x\, I\, (Gl)+\frac{4200}{95.5\, x\, 4}\, x\, I(CH_2)} \times 100\% \quad (S2)$$

$$C_{LA}(\text{wt.\%}) = \frac{144\, x\, I(La)}{162x\, I(H1)+\frac{114}{2}\, x\, I(\varepsilon)+144\, x\, I(La)+\frac{116}{2}\, x\, I\, (Gl)+\frac{4200}{95.5\, x\, 4}\, x\, I(CH_2)} \times 100\% \quad (S3)$$

$$C_{GL}(\text{wt.\%}) = \frac{\frac{116}{2}\, x\, I\, (Gl)}{162x\, I(H1)+\frac{114}{2}\, x\, I(\varepsilon)+144\, x\, I(La)+\frac{116}{2}\, x\, I\, (Gl)+\frac{4200}{95.5\, x\, 4}\, x\, I(CH_2)} \times 100\% \quad (S4)$$

$$C_{PEG}(\text{wt.\%}) = \frac{\frac{4200}{95.5\, x\, 4}\, x\, I(CH_2)}{162x\, I(H1)+\frac{114}{2}\, x\, I(\varepsilon)+144\, x\, I(La)+\frac{116}{2}\, x\, I\, (Gl)+\frac{4200}{95.5\, x\, 4}\, x\, I(CH_2)} \times 100\% \quad (S5)$$

Where; $C_{dex,\ Cap,\ LA,\ GL,\ PEG}$—percentage weight ratio of dextrin, caproil, lactidyl, glycolidyl units or polyethylene glycol, I(H1) - the values of the integrals of the dextrin's anomeric hydrogen, I(ε) - the values of the integrals of caproil ε C<u>H</u>$_2$ groups, I(LA) – the values of the integrals of lactidyl C<u>H</u> groups, I(Gl) – the value of integrals of glycolidyl C<u>H</u>$_2$ groups, I(CH_2) – the value of integrals C<u>H</u>$_2$ groups of PEG

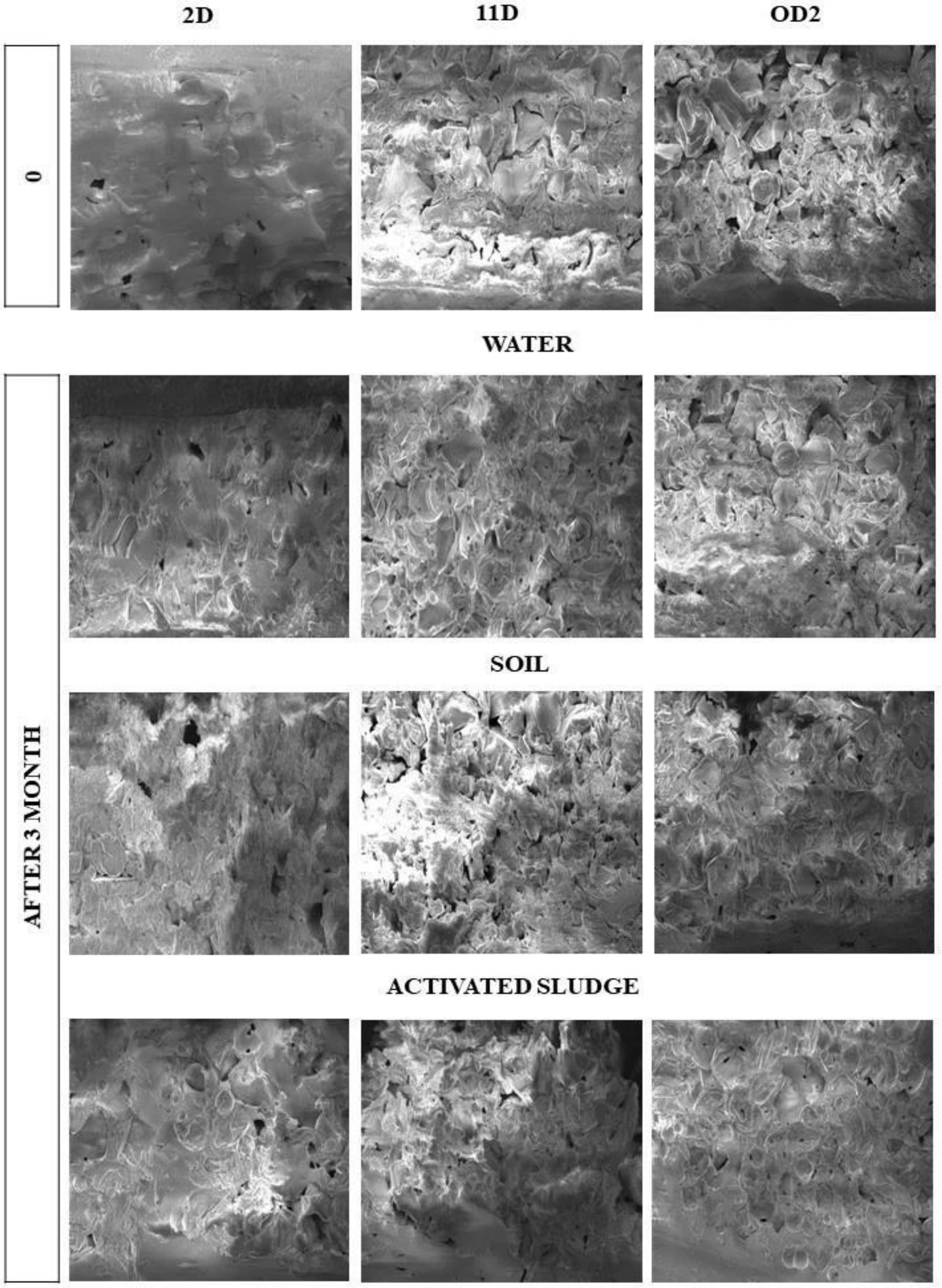

Figure 9S. SEM images of the degradation (after cross section, magnification 500μm) **2D** (LA/GA/PEG+D-g-PCL; R_{dex}=70 wt.%) 50:50; **11D** (LA/GA/PEG+D-g-PCL; R_{dex}=31 wt.%) 50:50 and **OD2** (LA/GA/PEG + D.UNMODIFIED 50:50 (100μm).

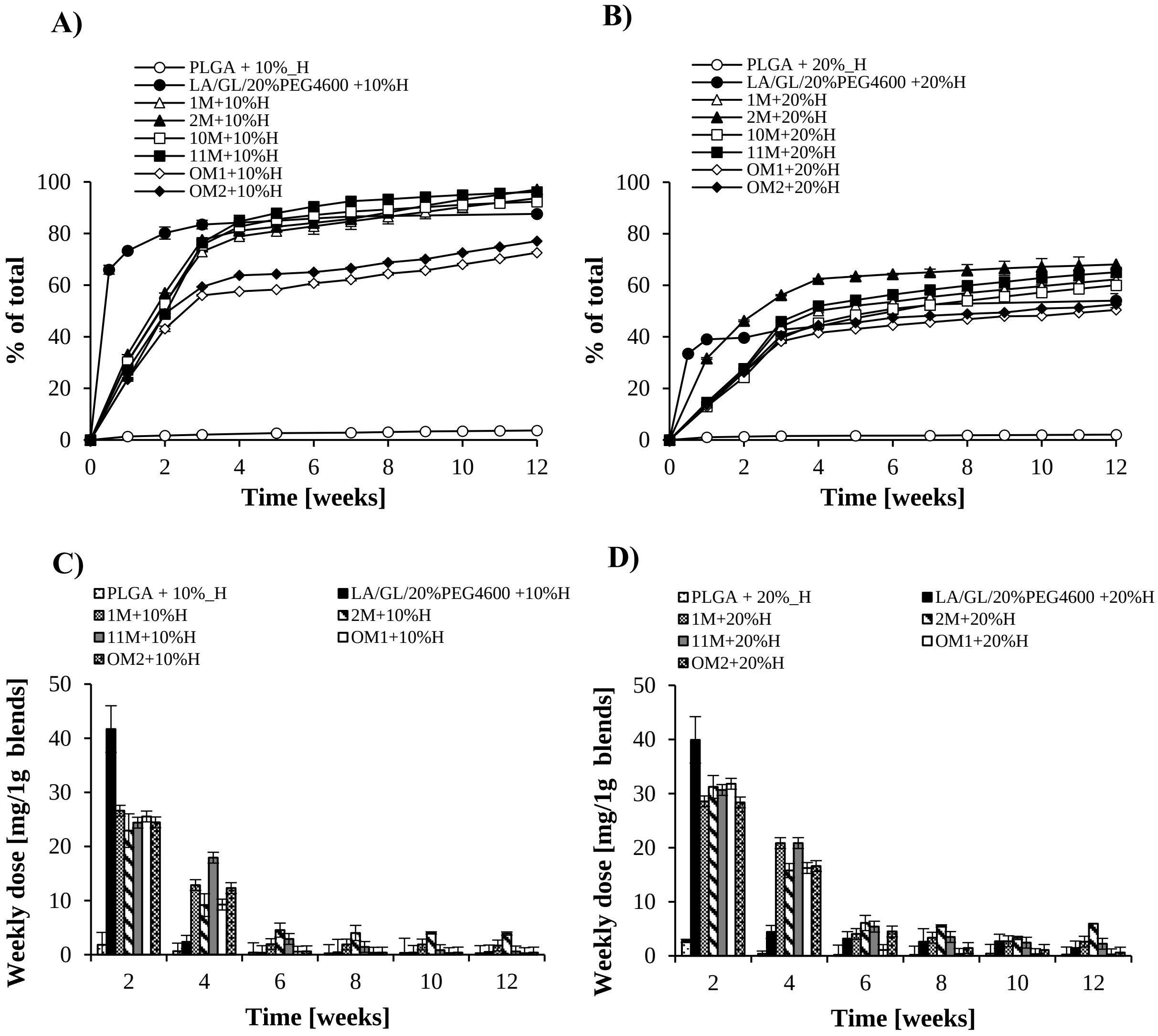

Figure 10S. Cumulative (A,B) and weekly dose release (C,D) of Metazachlor in water (maltodextrin).

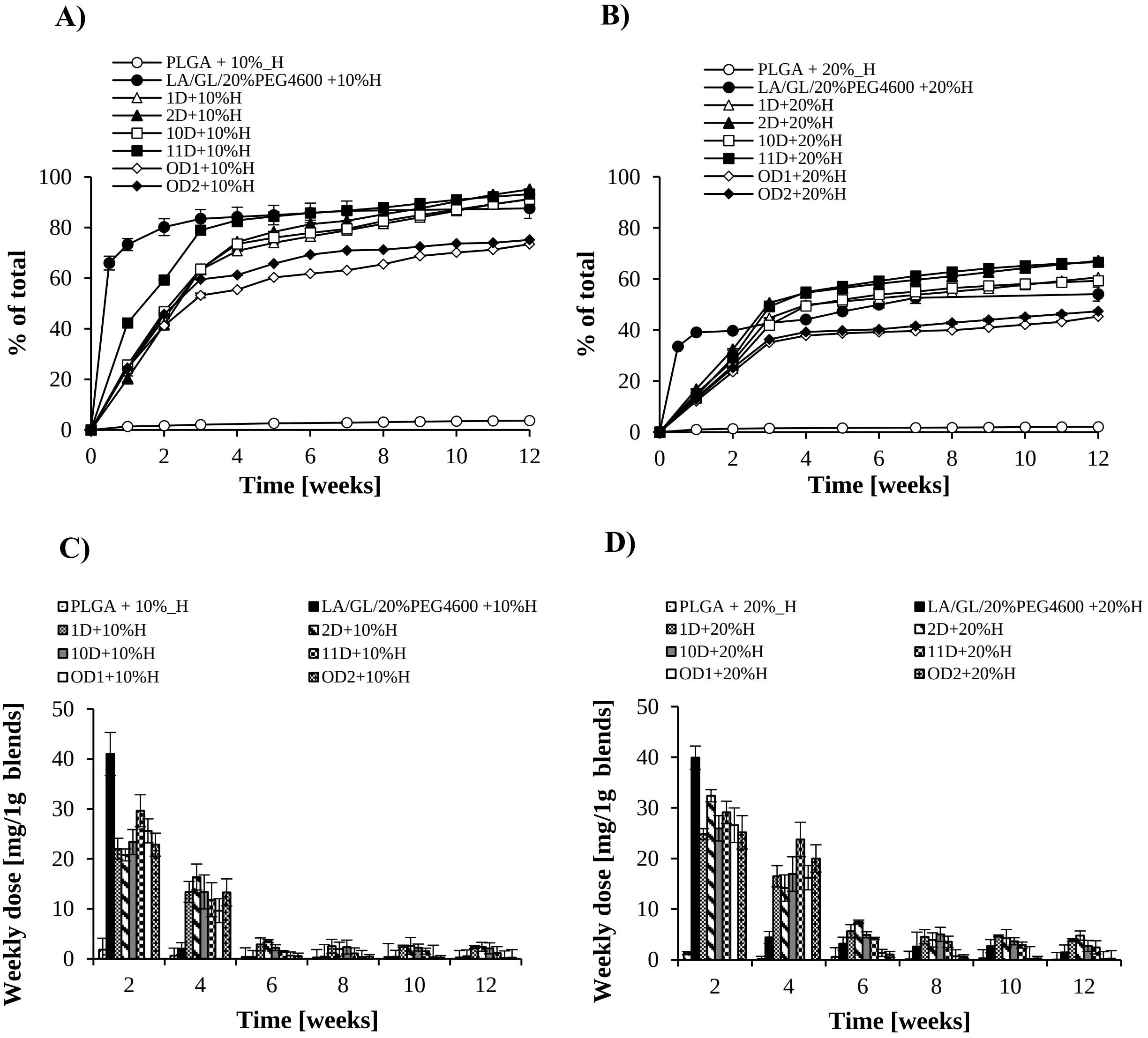

Figure 11S. Cumulative (A,B) and weekly dose release (C,D) of Metazachlor in water (dextrin).

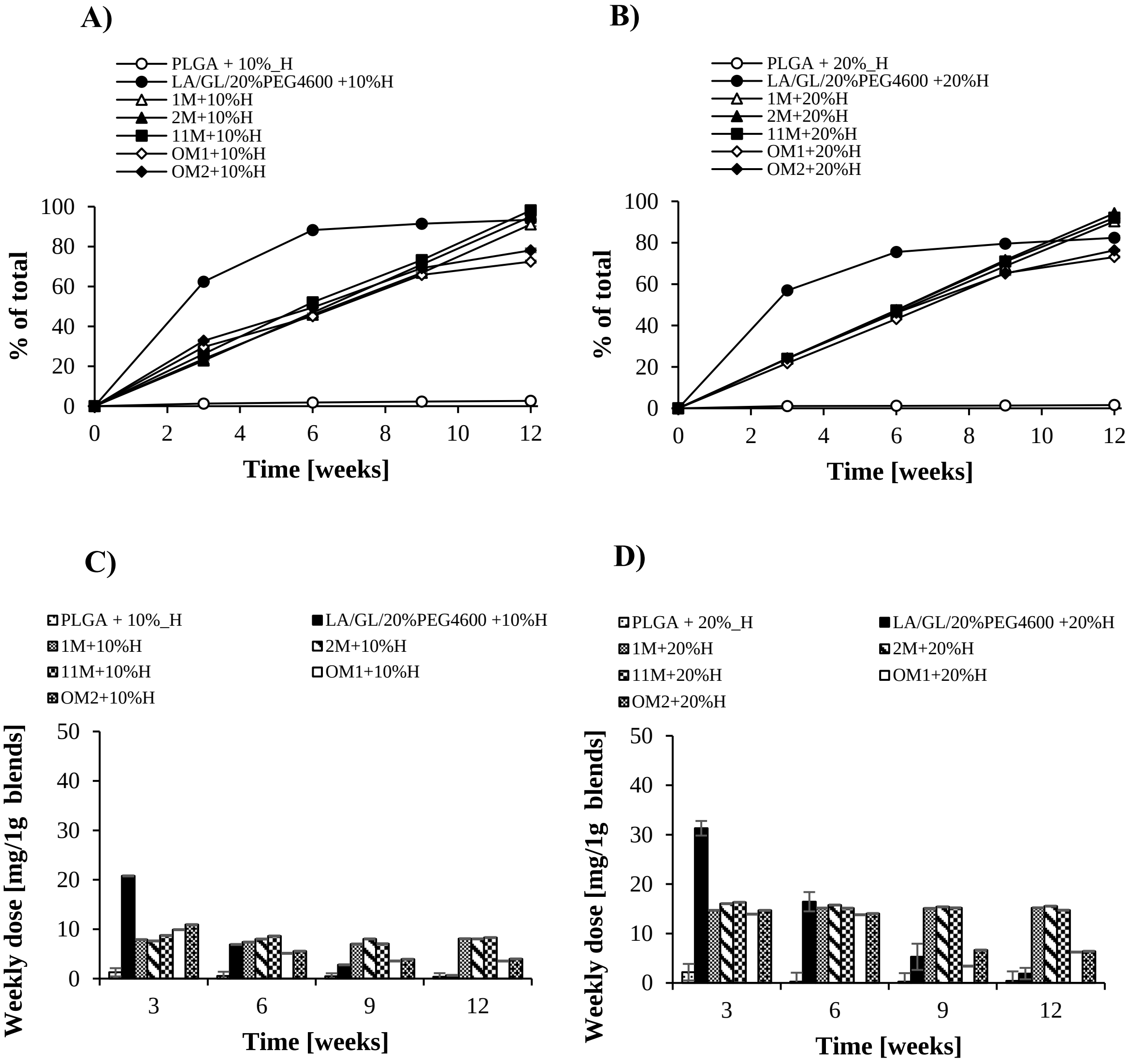

Figure 12S. Cumulative (A,B) and weekly dose release (C,D) of Metazachlor in soil (maltodextrin).

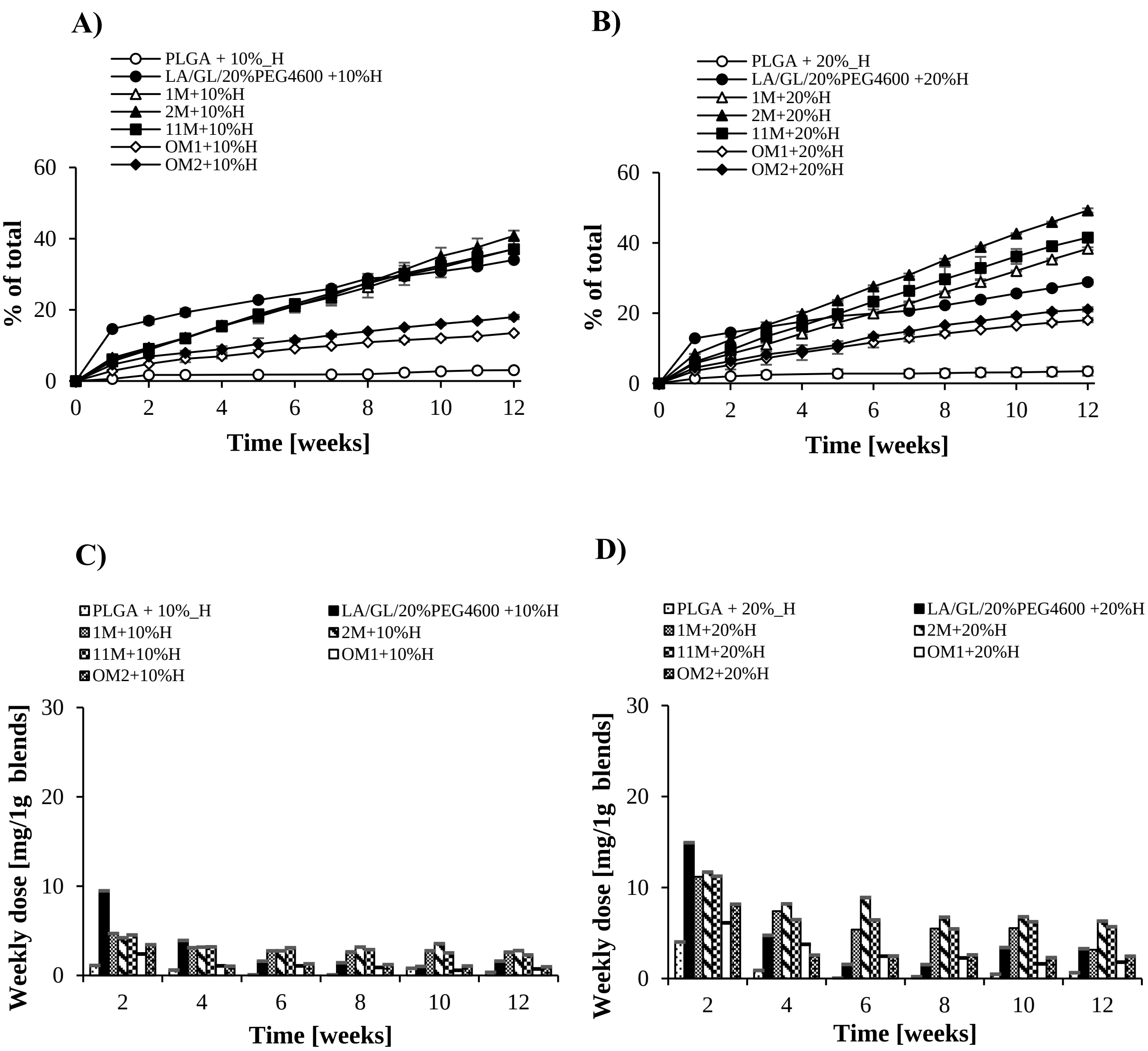

Figure 13S. Cumulative (A, B) and weekly dose release (C, D) of Pendimethalin in water (maltodextrin).

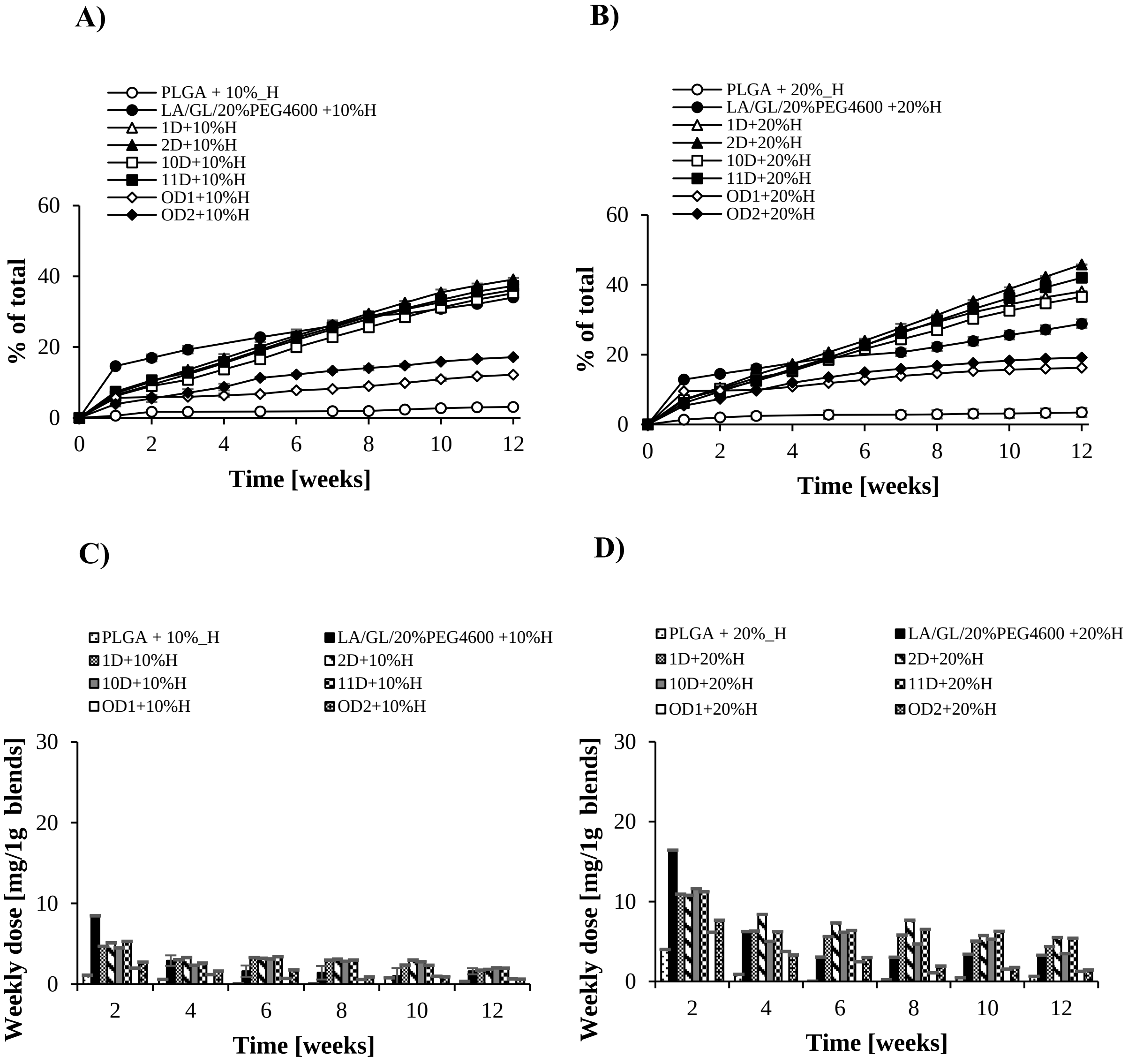

Figure 14S. Cumulative (A,B) and weekly dose release (C,D) of Pendimethalin in water (dextrin).

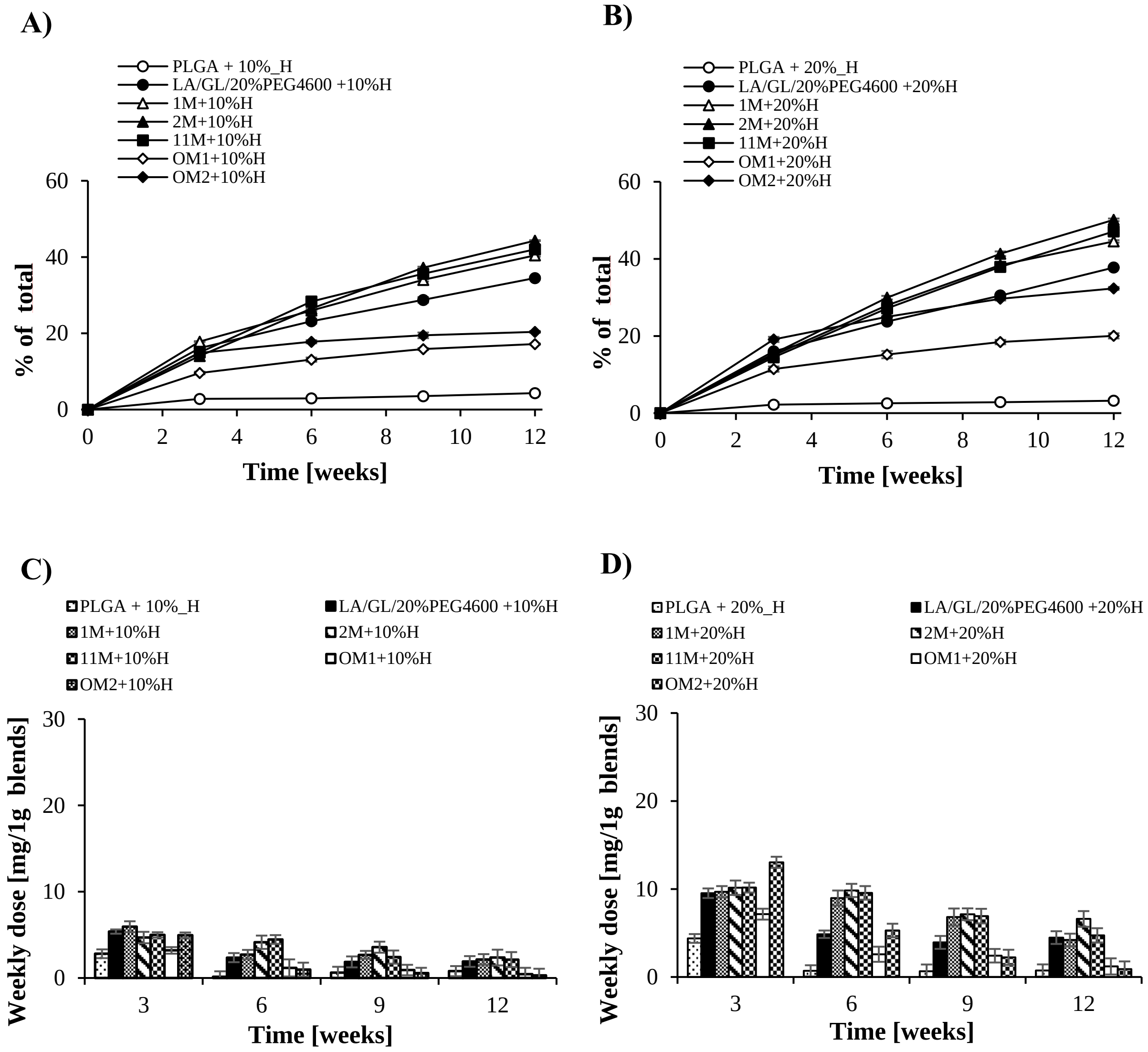

Figure 15S. Cumulative (A,B) and weekly dose release (C,D) of Pendimethalin in soil (maltodextrin).

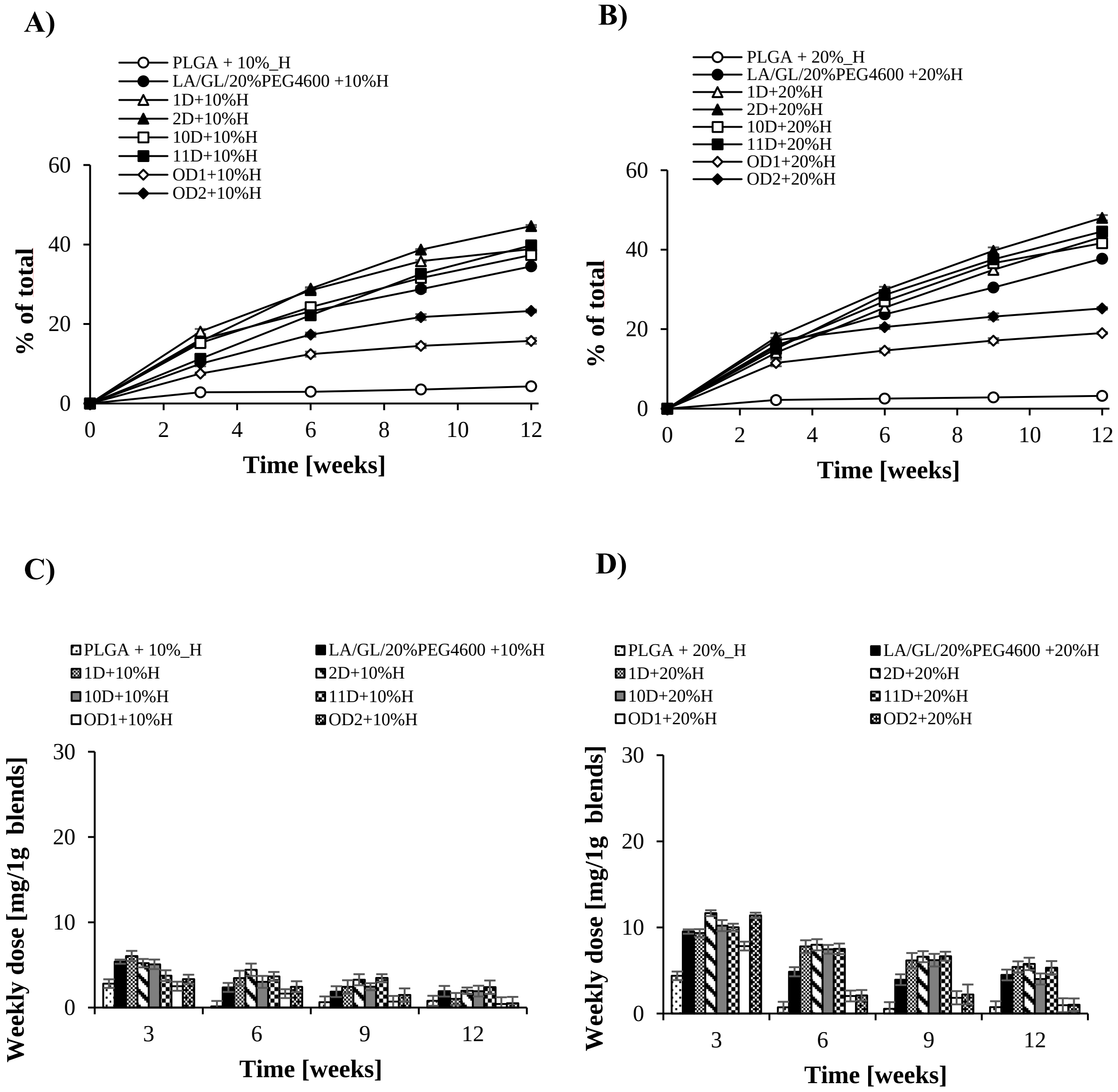

Figure 16S. Cumulative (A,B) and weekly dose release (C,D) of Pendimethalin in soil (dextrin).

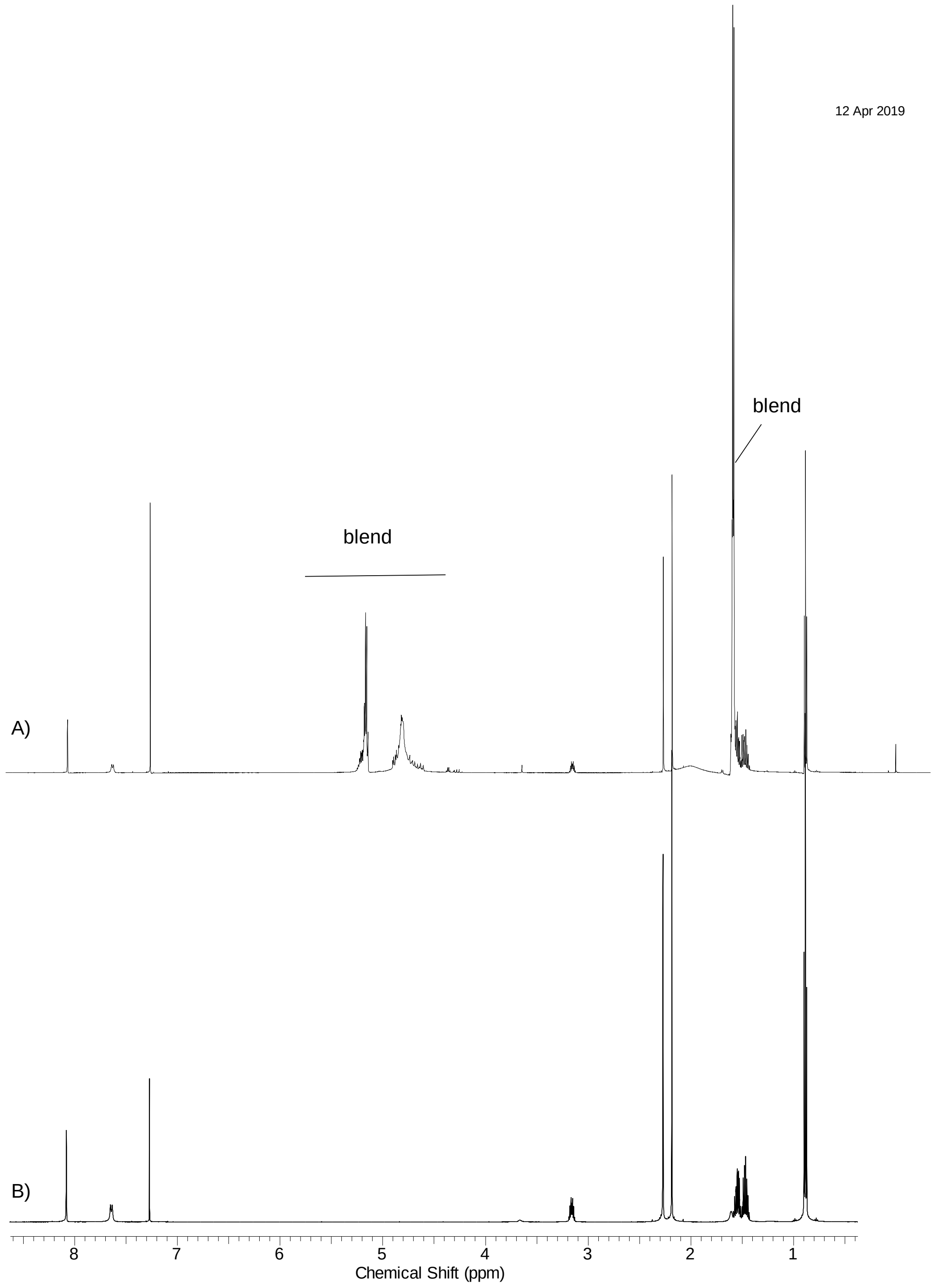

Figure 17S. 1H NMR spectra of (**A**) matrix containing Metazachlor residues after 12 weeks of release, (**B**) Metazachlor.

© 2020 by the authors. Submitted for possible open access publication under the terms and conditions of the Creative Commons Attribution (CC BY) license (http://creativecommons.org/licenses/by/4.0/).
